# *GNC* and *CGA1* Modulate Chlorophyll Biosynthesis and Glutamate Synthase (*GLU1/Fd-GOGAT*) Expression in *Arabidopsis*

**DOI:** 10.1371/journal.pone.0026765

**Published:** 2011-11-10

**Authors:** Darryl Hudson, David Guevara, Mahmoud W. Yaish, Carol Hannam, Nykoll Long, Joseph D. Clarke, Yong-Mei Bi, Steven J. Rothstein

**Affiliations:** 1 Department of Molecular and Cellular Biology, University of Guelph, Guelph, Ontario, Canada; 2 Syngenta Biotechnology Inc., Research Triangle Park, North Carolina, United States of America; Purdue University, United States of America

## Abstract

Chloroplast development is an important determinant of plant productivity and is controlled by environmental factors including amounts of light and nitrogen as well as internal phytohormones including cytokinins and gibberellins (GA). The paralog GATA transcription factors *GNC* and *CGA1/GNL* up-regulated by light, nitrogen and cytokinin while also being repressed by GA signaling. Modifying the expression of these genes has previously been shown to influence chlorophyll content in *Arabidopsis* while also altering aspects of germination, elongation growth and flowering time. In this work, we also use transgenic lines to demonstrate that *GNC* and *CGA1* exhibit a partially redundant control over chlorophyll biosynthesis. We provide novel evidence that *GNC* and *CGA1* influence both chloroplast number and leaf starch in proportion to their transcript level. *GNC* and *CGA1* were found to modify the expression of chloroplast localized *GLUTAMATE SYNTHASE* (*GLU1/Fd-GOGAT*), which is the primary factor controlling nitrogen assimilation in green tissue. Altering *GNC* and *CGA1* expression was also found to modulate the expression of important chlorophyll biosynthesis genes (*GUN4*, *HEMA1*, *PORB*, and *PORC*). As previously demonstrated, the *CGA1* transgenic plants demonstrated significantly altered timing to a number of developmental events including germination, leaf production, flowering time and senescence. In contrast, the *GNC* transgenic lines we analyzed maintain relatively normal growth phenotypes outside of differences in chloroplast development. Despite some evidence for partial divergence, results indicate that regulation of both *GNC* and *CGA1* by light, nitrogen, cytokinin, and GA acts to modulate nitrogen assimilation, chloroplast development and starch production. Understanding the mechanisms controlling these processes is important for agricultural biotechnology.

## Introduction

Improving agricultural productivity is essential for maintaining global development and necessary in order to permit future population growth [1]. Historical increases in plant productivity achieved through irrigation, fertilizer application, hybrid selection or genetic modification can be largely attributed to a crops ability to maximize photosynthetic capture [2]. Differences in chlorophyll content and/or chloroplast number are typically directly related to agricultural productivity, with greener plants having increased nitrogen use efficiency, biomass and yield [2], [3]. Plastids, including chloroplasts, are determined early in the plant meristem and further differentiation occurs according to the type of cell in which they will ultimately reside [4]. Still, the actual number of chloroplasts in a mature leaf cell as well as the abundance of pigments within each chloroplast depends on both the developmental stage of particular tissues and environmental stimuli [5], [6]. Plants must use overlapping networks to coordinate chloroplast development with a plethora of environmental inputs in order to maintain balance between rates of photosynthesis and metabolism.

Light amount and quality are powerful regulators of chlorophyll biosynthesis and chloroplast development. Light also establishes circadian and diurnal cycles that provide a constant internal control over gene expression and when in tune with environmental signals, plants display maximum growth [7]–[9]. Nitrogen is required for building biological molecules and is therefore also intrinsically linked to both photosynthetic activity and the overall carbon status of the plant [10], [11]. Nitrogen assimilation in the chloroplast is a prerequisite for chlorophyll biosynthesis, specifically by building up the glutamate pool [12], [13]. The Glutamine Synthetase/Glutamate Synthase (GS/GOGAT) pathway is a key point in nitrogen assimilation where ammonium is incorporated into glutamate, providing the precursor for production of all amino acids, nucleic acids and chlorophylls [13], [14]. The subsequent steps involved in chlorophyll biosynthesis are well documented and involve a number of key rate-limiting enzymes [15]. *HEMA1* encodes a Glu-tRNA reductase enzyme that controls flux through the tetrapyrrole biosynthetic pathway and leads to production of 5-aminolevulinic acid (ALA) from which the porphyrin ring system is derived [16], [17]. *GENOMES UNCOUPLED 4 (GUN4)* subsequently binds protoporphyrin chlorophyll intermediates (Mg-Proto and Mg-ProtoMe), stimulates Mg chelatase activity, and has also been implicated in plastidic retrograde signaling to regulate nuclear gene expression [18]–[20]. Light-dependent reduction of protochlorophyllide to chlorophyllide is catalyzed by NADPH∶protochlorophyllide oxidoreductase (POR) in mature leaves, where the genes *PORB* and *PORC* form a redundant system regulating chlorophyll biosynthesis [21]. Whilethese genes demonstrate circadian and diurnal patterns of expression [15], [22], the exact mechanism by which chlorophyll content is adjusted with varying amounts of light and nitrogen is not well documented.

Because nitrogen is a key component of the chlorophyll molecule, the concentration of nitrate available to a plant directly influences chlorophyll biosynthesis and chloroplast development [23], [24]. Chlorophyll content is a key indicator of plant health and can be used to optimize nitrogen fertilizer application in order to potentiate larger crop yields with lower environmental load [25], [26]. A subset of nitrate responses are mediated by the class of plant hormones know as cytokinins, whose synthesis and transport is linked to the nitrogen status of the plant [27]–[29]. Cytokinin signaling plays a central role in the regulation of cell division, differentiation and various developmental processes including chlorophyll biosynthesis and chloroplast development [30], [31]. Cytokinins have also been shown to exert control over the process of N-remobilization and grain development [31]–[34]. Understanding the processes involved in fine tuning chloroplast development and grain production with fluctuating light and nitrogen levels is vital for making agricultural improvements in crop plants.

Cytokinins exhibit antagonistic effects to another class of plant hormones known as Gibberellins (GA). In *Arabidopsis*, cytokinin and GA signaling exert opposing influences over organ size, chlorophyll levels and floral development [35]–[38]. The GA-mutants largely responsible for the Green Revolution demonstrate that signaling reducing chloroplast development is just as important as factors promoting it [39]. GA repression of chlorophyll biosynthesis occurs through the activity of DELLA proteins and phytochrome-interacting factors (PIF's). DELLA proteins mediate transduction of GA signals to light-responsive transcription factors, derepressing chlorophyll and carotenoid biosynthetic pathways though interactions with PIFs [40], [41]. DELLA's were recently shown to play a crucial role in the formation of functional chloroplasts during deetiolation [41]. PIFs are a class of the basic helix-loop-helix family of transcription factors that function in nuclear protein interaction cascades as negative regulators of phytochrome mediated light responses [40]. In the absence of GA, nuclear-localized DELLA proteins accumulate to higher levels and prevent PIF's, from binding to their target promoters [40]. The presence of GA triggers DELLA degradation, thus releasing PIF's to function and subsequently repressing genes involved in chlorophyll biosynthesis [22], [40], [41]. This results in a complicated system of regulation for chlorophyll biosynthesis genes where the established circadian control of gene expression is subject to further influence from light, nitrogen, cytokinin and GA signaling pathways.

The paralog GATA transcription factors *GNC (GATA*, *NITRATE-INDUCIBLE*, *CARBON-METABOLISM-INVOLVED)* and *CGA1/GNL (CYTOKININ-RESPONSIVE GATA 1/GNC-LIKE)* are excellent candidates for integrating the signals involved in chloroplast development [42]–[44]. These paralogs display high levels of expression in light-grown plants, are strongly induced by light following periods of darkness in a phytochrome-dependent manner [31], [45] and are regulated in a circadian fashion with peak transcript abundance at pre-dawn [46]. Both *GNC* and *CGA1* transcripts are induced by nitrogen sources [42], [46], [47] as well as by cytokinins [43], [48], [49]. Altering the expression levels of both *GNC* and *CGA1* has previously been shown to influence chlorophyll content in *Arabidopsis* [38], [42], [44]. Systems biology has been used to predict that *GNC* and *CGA1* act in a biological network with important chloroplast localized genes [50]. It was recently confirmed that altering the expression of *GNC* and *CGA1* results in reduced expression of the *POR* genes involved in chlorophyll biosynthesis [38]. *GNC* and *CGA1* transcript levels were also shown to be suppressed by GA signaling through the activity of DELLA proteins and PIF3 [38]. Furthermore, *GNC* and *CGA1* expression is altered in GA signaling and *pif* mutants, which could partially explain the differences in chlorophyll observed in these lines [38], [51]. In addition to changes in chlorophyll biosynthesis, transgenic alterations *GNC* and *CGA1* expression were shown to influence germination, expansion growth and flowering time [38]. This further suggests that *GNC* and *CGA1* play an important role in maintaining the balance between GA and cytokinin signaling.

In this work, we provide evidence for regulation of the chloroplast localized *GLUTAMATE SYNTHASE* (*GLU1/Fd-GOGAT*) by both *GNC* and *CGA1*, as well as confirm their role in modulating the expression of genes involved in chlorophyll biosynthesis, including *HEMA1*, *GUN4 PORB* and *PORC*. We demonstrate that changing the expression of *GNC* or *CGA1* leads to changes in chloroplast development, modulating not only chlorophyll content, but also chloroplast number and total leaf starch. Like Richter et al. [38], *CGA1* expression was found to have a significant influence over the timing of important developmental events including germination, flowering time and senescence, which indicates an additional role in modulating crosstalk between cytokinin and gibberellin signaling. However, we did not observe significant differences in the timing of these developmental events with altered *GNC* expression. Despite this evidence of partial divergence, our results support the theory that both *GNC* and *CGA1* integrate signals from light, nitrogen, cytokinin, and GA in order to modulate nitrogen assimilation and chloroplast development in a partially redundant fashion.

## Results

### *GNC* Influences Chlorophyll Content, but does not Directly Control Sugar Signaling

**Figure 1:**
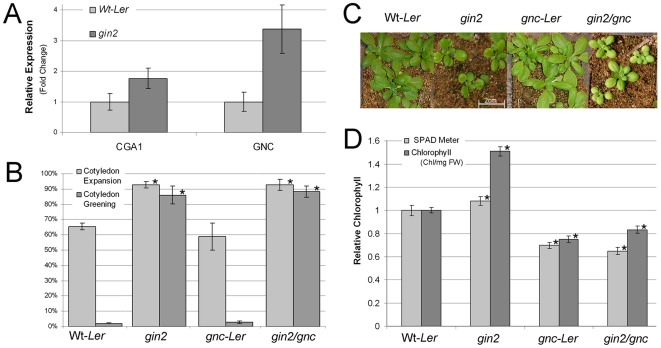
*GNC* inlfuences chlorophyl content but does not directly regulate sugar signalling or transport. A) Expression levels of *CGA1* and *GNC* in the *HEXOKINASE1* mutant (*gin2)* using *q*RT-PCR from 3 week old rosette leaf tissue. B) Cotyledon expansion (where cotyledons open but appear pale and etiolated) and cotyledon greening (where expanded cotyledons also produce chlorophyll) of transgenic lines compered to wild-type on high glucose (6%) MS media. C) Images of 3 week old Wt-*Ler*, *gnc-Ler*, *gin2* and *gin2/gnc* plants. D) Chlorophyll content measured with the standard acetone extraction and with the Minolta SPAD 502DL meter. Due to the decreased leaf and cell size of the *gin2* mutant, using the acetone based extraction technique that controls for biomass requires a larger number of leaves/cells to be harvested for comparative analysis. Results taken with the SPAD meter are nondestructive and use light transmitted through a single leaf. Both techniques demonstrate that the *gin2/gnc* double mutant lacks chlorophyll accumulation, though the SPAD readings show less variation and are not as influenced by overall differences in plant size (All data MEAN±SD, * indicates p≤.05, t-test).

*GNC* was originally predicted to have connections to sugar sensing and signaling [42]. The expression of *Hexokinase1* (*HXK1*) involved in glucose sensing, signaling and phosphorlyation is increased in *gnc* and *cga1* mutants [42], [44]. To investigate the relationship between *GNC* and sugar signaling further, we used the *glucose insensitive 2* line (*gin2-1*), which is a null mutation for *HXK1* [52], [53]. The *gin2* mutation causes insensitivity to growth inhibition on high sugar media, insensitivity to auxin and hypersensitivity to cytokinin [52], [53]. The *gin2* line has increased greening but decreased cell expansion and adult plants are less than half the size of Wt-*Ler (Landsberg)* controls [53], [54]. Since the expression of *GNC* and *CGA1* is altered in GA signaling mutants (*ga1*, *gid1abc*) that show changes in chlorophyll content [38], we questioned whether their transcripts were also expressed differently in other mutants altering chlorophyll content such as *gin2*. *GNC* transcript levels were found to be increased (∼3 fold) in the *gin2* mutant and *CGA1* expression was also slightly enhanced, though to a lesser extent (Figure 1A). Increased *GNC* and *CGA1* expression may at least in part account for the increased chlorophyll of the *gin2* line.

To investigate this further, we created a *gnc/gin2* double mutant. The SALK_*01778-gnc* mutant (*Columbia* background) was back-crossed into the Wt-*Ler* ecotype for five generations in order to produce a *gnc-Ler* mutant. As in *Columbia*, the only obvious phenotype in the *gnc-Ler* mutant was reduced chlorophyll content (Figure 1C and D). Reciprocal crosses between *gin2* and *gnc-Ler* produced *gin2/gnc* double mutants. Progeny of stable homozygous *gin2/gnc* plants were plated on 6% glucose (Figure 1B). The *gnc/gin2* double mutants exhibited similar insensitive growth characteristics to *gin2* single mutants on high glucose media (Figure 1B). In contrast, wild type and *gnc* mutant plants exhibited similar sugar sensitive responses and demonstrated inhibition of both cotyledon expansion and greening (Figure 1B). Chlorophyll was extracted from 3 week old plants by using the standard acetone based extraction technique that controls for biomass as well as measured nondestructively with the Minolta SPAD-501 meter (Figure 1D). Plants homozygous for the *gnc* mutation exhibit decreased chlorophyll content, removing the dark green phenotype present in the *gin2* line (Figure 1C and D). The sugar insensitivity and lack of chlorophyll in the *gin2/gnc* double mutants indicates that *GNC* is epistatic to *HXK1* with respect to chlorophyll biosynthesis, but does not appear to directly regulate *HXK1*-dependent sugar signaling.

### *GNC* and *CGA1* Regulate Chlorophyll Production in the *ahk2/3* Cytokinin Receptor Mutant

**Figure 2:**
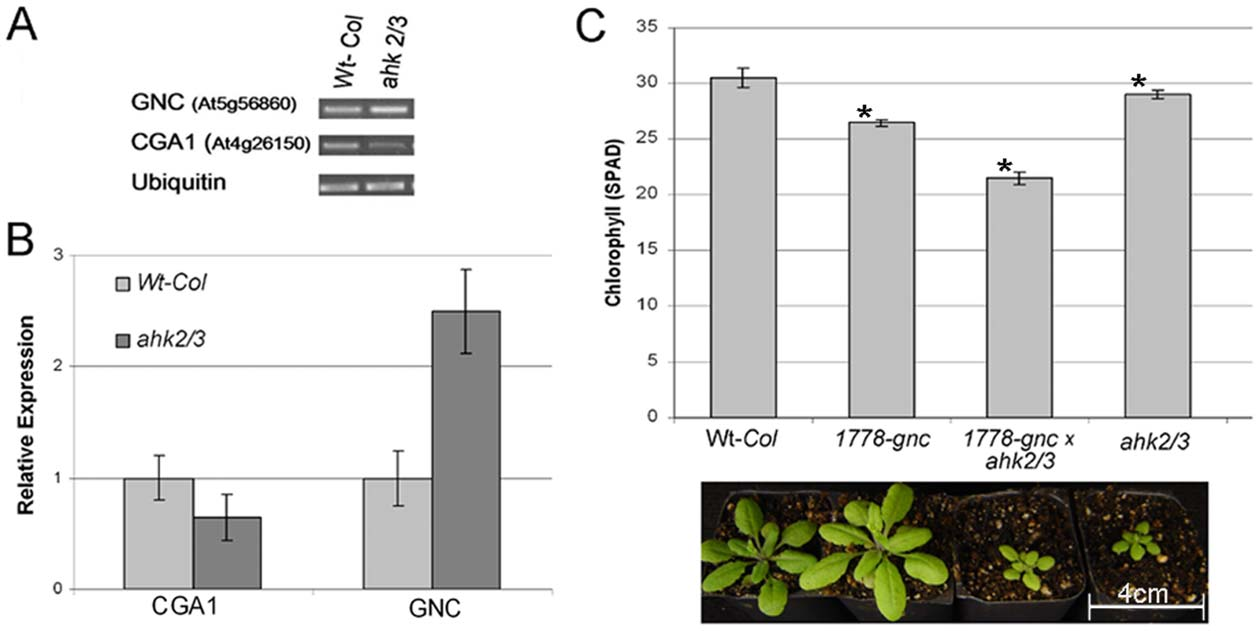
*GNC* and *CGA1* expression alters chlorophyll content in the *ahk2/3* cytokinin receptor mutant. Both semi-quantitive PCR (A) and Real-time *q*RT-PCR (B) demonstrate that *CGA1* is down regulated in the leaves of the *ahk2/3* cytokinin receptor mutant while *GNC* expression is slightly increased. C) Chlorophyll content of *1778-gnc*, *ahk2/3* and *gnc/ahk2/3* mutant lines compared to wild type (Wt-*Col*) plants at 3 weeks post germination (MEAN±SD, p≤.05, t-test). Image demonstrates that the triple mutant is of a similar size to *ahk2/3* double mutant, but also has drastically reduced chlorophyll content.

The spatial expression profiles of *GNC* and *CGA1* [42]–[44], [55] demonstrate similar patterns as the cytokinin receptors *AHK2* and *AHK3* [55], [56]. They are also both up-regulated by cytokinin application, though *CGA1* transcript levels increase more rapidly and fluctuate to a greater extent [43], [48]. The *ahk2/3* double mutant has significantly reduced chlorophyll content [57] and was analyzed for expression of *GNC* and *CGA1*. *CGA1* expression was found to be decreased, in the *ahk2/3* mutant (Figure 2A and B). In contrast, we found that *GNC* expression was up-regulated in the *ahk2/3* double mutant (Figure 2A and B). To investigate this further, crosses were made between the *gnc* mutant and the *ahk2/3* cytokinin receptor double mutant to create a *ahk2/3/gnc* triple mutant line. The resulting *ahk2/3/gnc* triple mutant had significantly reduced chlorophyll content when compared to all other lines (Figure 2C). Combining the reduced expression of *CGA1* found in the *ahk2/3* mutant with a mutation of *gnc* caused further reductions in chlorophyll. Beyond the drastically reduced chlorophyll in the *ahk2/3/gnc* plants, we did not observe significant phenotypic differences compared to *ahk2/3* mutants. These results imply that *GNC* acts specifically to control chlorophyll biosynthesis and functions in a partially redundant fashion as *CGA1*.

### *CGA1* Expression Influences Developmental Timing in *Arabidopsis* in a Manner Consistent with Altered Cross-talk between Cytokinin and Gibberellins Signaling

**Figure 3:**
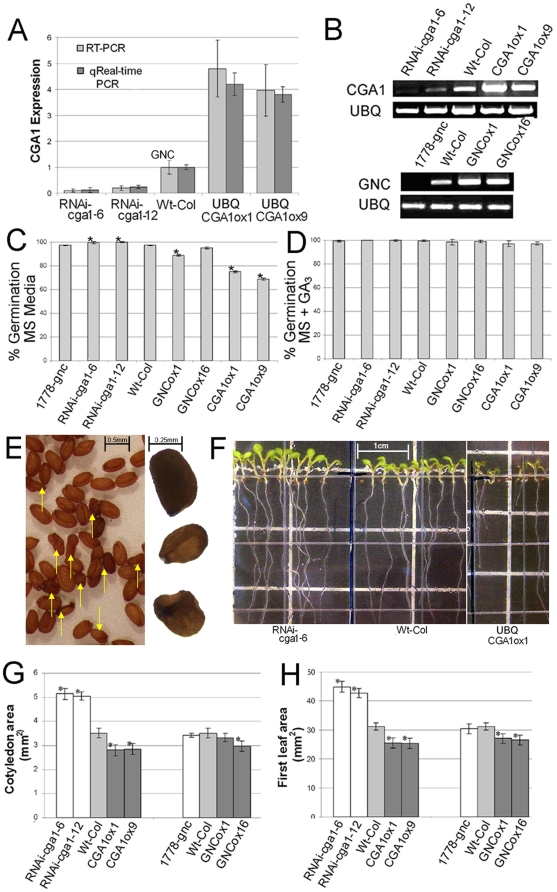
Early developmental analysis of *CGA1* transgenics indicates influence over germination and leaf expansion. A) Relative expression level of *CGA1* in the transgenic lines created for this study. *q*RT-PCR on extracts from rosette leaves of positively transformed 3 week old plants. B) Semi-quantitative RT-PCR on progeny of trangeneic plants (9 pooled plants) showing stable and transmissible expression levels in subsequent generation. C) Germination of transgenic *GNC* and *CGA1* lines on MS media. D) Germination of transgenic *GNC* and *CGA1* lines on MS media containing gibberellin (3 µM GA_3_). E) Seed produced from *CGA1* overexpression lines. Yellow arrows point to seeds with deformed seed coats (right). Magnified image (left) showing one normally developed seed (top) and two smaller seeds with deformed seed coats. F) Seedlings from *CGA1* transgenic lines grown for one week on vertical MS media. G) Cotyledon surface area from one week old plants grown on soil. G) First leaf surface area from two week old plants. (All data MEAN±SD, p≤.05, t-test).

In this study, we used the following genetic lines with altered levels of *GNC* and *CGA1* expression. The two 35S∶*GNC* overexpression lines (*GNC*ox1 and *GNC*ox16) with near 4-fold increases in *GNC* transcript levels and the *SALK01778-gnc* mutant were established in our previous work [42]. Our attempts to identify a true mutation for *CGA1* from publicly available T-DNA insertion lines (eg. SALK_03995 and SALK_0213625) proved unsuccessful [42]. As such, RNAi driven by an endogenous ubiquitin (*UBQ*) promoter was used to significantly reduce the expression of *CGA1* to 10–20% of wild type in both lines analyzed (*RNAi-cga1-6 and RNAicga1-12*) (Figure 3A). For overexpression, two constitutive *UBQ∶CGA1* lines (CGAox1 and *CGA1*ox4) were also created, which like the *GNCox* lines tested [42], had an approximate 4-fold increase in transcript level compared to wild type controls (Figure 3A). For all the lines analyzed, transcript expression levels were stable in subsequent generations (Figure 3B).

Similar to the results recently reported by Richter et al. [38], we found that expression of *CGA1* significantly influences a number of developmental events in *Arabidopsis*. *CGA1* transgenic plants exhibit phenotypes similar to those seen with altered GA signaling [36], [38], [58], [59]. GA is known to influence germination, chlorophyll content, stem elongation, flowering time and senescence [36], [40]. Altering *CGA1* expression also results in differences in germination with nearly 100% of *RNAi-cga1* seed germinating, while *CGA1* overexpression reduced or delayed germination (Figure 3C and F). *RNAi-cga1* plants produced seed that looked normal compared to wild-type plants; however, typically between 18–22% (20.6%±2.1, MEAN±SD) of the seed from *CGA1* overexpression lines did not set properly, resulting in seeds with deformed seed coats that were smaller in size (Figure 3E). GA has been shown to significantly influence seed dormancy and germination as well as contribute to formation of the seed coat through starch degradation [40], [60], [61]. The addition of GA_3_ to MS media removed the low germination of the *CGA1* overexpression lines and allowed the deformed seeds to germinate (Figure 3D). Like Richter et al. [38], we also found that following germination, expansion and overall size of seedlings was also significantly different in *CGA1* transgenics. *RNAi-cga1* plants are visibly larger after one week, whereas overexpression lines are smaller than wild type plants (Figure 3F). Both cotyledon and first leaf size were found to be inversely proportional to the level of *CGA1* transcript (Figure 3G and H).

**Figure 4:**
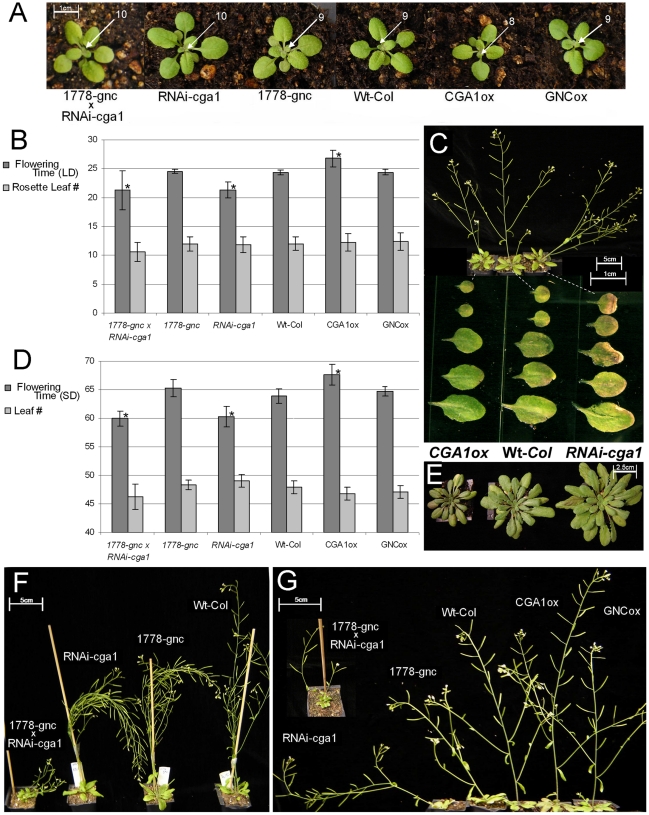
Phenotypic analysis of plants with altered expression of *GNC* and *CGA1*. A) Images of pre-flowering plants with arrows showing the newest leaf extended beyond 1 mm indicate the rate of leaf production is altered in *CGA1* transgenic lines. B) Flowering time and rosette leaf number at flowering of transgenic plants compared to wild-type grown under long day (LD) conditions (MEAN±SD, p≤.05, t-test). C) Altered rates of leaf senescence in mature *CGA1* transgenics indicate differences in growth rate through the entire life cycle (LD). D) Flowering time and leaf number at the time of flowering for transgenic plants grown under short day (SD) conditions (MEAN±SD, p≤.05, t-test). E) Difference in size of pre-flowering *CGA1* plants compared to Wt- *Col* grown in SD. F) Reduced expression of *GNC* or *CGA1* results in spindly stems defective in standing upright when grown at sufficient nitrogen levels (10 mM NO_3_^−^, LD). G) Transgenics GATA lines grown at limiting nitrogen conditions (3 mM NO_3_^−^, LD) show even greater differences in stem integrity.

**Figure 5:**
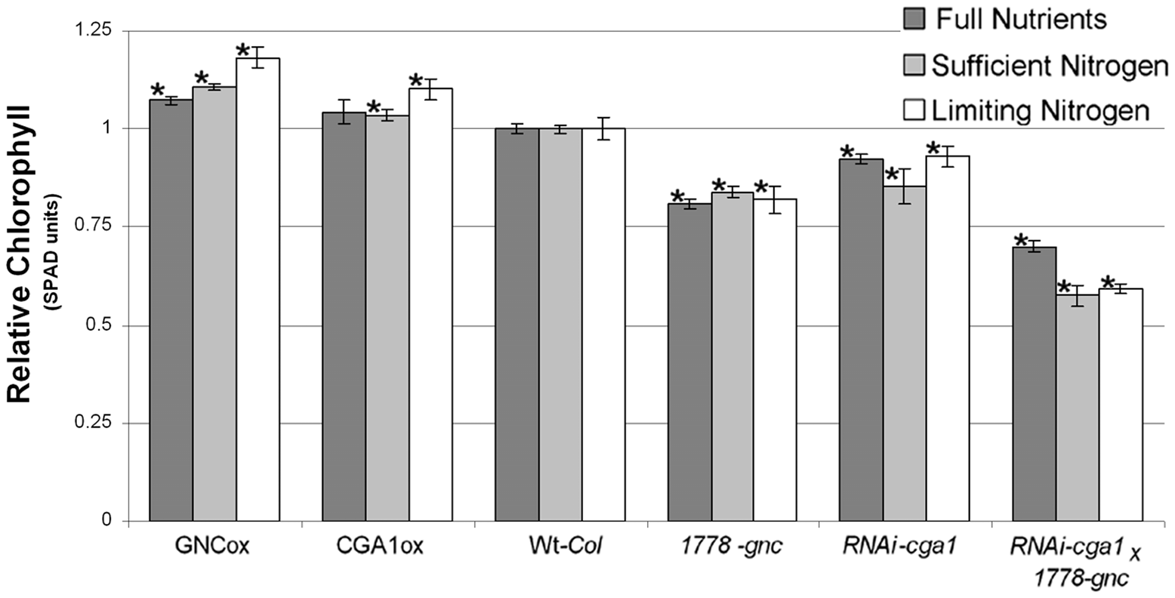
*GNC* or *CGA1* expression alters chlorophyll content. Relative chlorophyll content of GNC and CGA1 transgenics compared to wild type controls. Plants grown with high light and full nutrients (300 µmol/m^2^-s light and excess nutrient fertilizer), sufficient nitrogen (150 µmol/m^2^-s light and 10 mM NO_3_^−^) and limiting nitrogen (150 µmol/m^2^-s light and 3 mM NO_3_^−^) all demonstrate differences in chlorophyll SPAD (MEAN±SD, p≤.05, t-test).

Reciprocal crosses were made between the *RNAi-cga1* lines and the *gnc* mutant to create pseudo-double mutants used in subsequent analysis. Though the *RNAi-cga1 × gnc* mutant plants demonstrated further reductions in chlorophyll compared to single mutants (Figures 4 and 5), they developed at a similar rate as the *RNAi-cga1* plants (Figure 4). At 18 days post-germination, both the wild type and *GNC* lines had extended their 9^th^ leaf beyond 1 mm; in contrast, the *RNAi-cga1* and double mutant plants had already produced their 10^th^ leaf, while *CGA1*ox plants had only produced 8 leaves (Figure 4A). Both *RNAi-cga1* and the *RNAi-cga1*x *gnc* mutant plants exhibited early flowering with the inflorescence bolting approximately 3 days prior to wild type plants under long days (LD), while *CGA1* overexpression delayed flowering time by a similar interval (Figure 4B). Flowering was altered similarly under both long and short day (SD) conditions in the *CGA1* transgenics without drastically influencing rosette leaf number at flowering time (Figure 4B and D). Plants with reduced *CGA1* expression germinate earlier, produced leaves faster, flowered earlier and senescence more rapidly under both LD and SD conditions, while *CGA1* overexpression delays germination, growth, flowering and senescence irrespective of day length (Figure 4D and E). These results indicate that *CGA1's* influence is not specific to any one developmental event, but rather alters the rate of progression through the entire life cycle. As observed by Richter et al. [38], *GNC* overexpression lines demonstrated some evidence of reduced germination and expansion during early development. However, we found that neither mutation nor overexpression of *GNC* resulted in significant phenotypic differences from wild type plants with respect to overall growth rate, flowering time or senescence (Figure 4).

In previous studies, we established nutrient conditions where 3 mM nitrate was found to be a limiting nitrogen condition, under which effects of nitrogen stress can be observed, while 10 mM nitrate is sufficient to maintain normal growth and development in *Arabidopsis* [23], [42]. Under both sufficient and limiting nitrogen conditions, *RNAi-cga1* plants produced ‘spindly’ stems (Figure 4F and G). Though not as severe, this phenotype was also observed in the *gnc* mutant and the *RNAi-cga1 × gnc* plants were especially deficient in producing strong inflorescences (Figure 4F and G). Mutation of the N-acetlyglucosamine transferase gene named *SPINDLY* (*SPY*) results in a similar phenotype due to improper regulation of the cross-talk between cytokinin and GA signaling [35], [62]. Under limiting nitrogen conditions, this spindly phenotype was further exacerbated and the *RNAi-cga1*x *gnc* mutants produced very thin stems with few flowers and seeds (Figure 4G). In contrast, both *GNC* and *CGA1* overexpression lines produced strong inflorescences that maintained their erect stature even under limiting nitrogen conditions (Figure 4G). Phenotypic results suggest that *GNC* and *CGA1* have both partially redundant and partially divergent roles in regulating the balance between GA and cytokinin signaling pathways.

### *GNC* and *CGA1* Expression Influences Chlorophyll Content, Chloroplast Number and Leaf Starch

Both mutant and overexpression lines of *GNC* and *CGA1* result in modifications to chlorophyll biosynthesis [38], [42], [44]. Growing the transgenic lines under various light and nitrogen treatments resulted in further differences in chlorophyll content (Figure 5). Under all the conditions we analyzed, *gnc* mutants, *RNAi-cga1* and the *RNAi-cga1 × gnc* plants show significant reductions in chlorophyll content compared to wild type plants (Figure 5). Similar to the reported *cga1* mutants [38], [44], *RNAi-cga1* plants were not as pale as the *gnc* mutant. However, further reductions in the *RNAi-cga1 × gnc* plants imply partially redundant control over chlorophyll biosynthesis by these paralogs. Double mutants retain only 50–60% of wild type chlorophyll content depending on the levels of light and nitrogen supplied (Figure 5). While both wild type and transgenic lines show lower overall chlorophyll content with reduced nitrogen, *GNC* and *CGA1* overexpression lines maintain higher chlorophyll relative to wild type controls when nitrogen is limiting (Figure 5).

**Figure 6:**
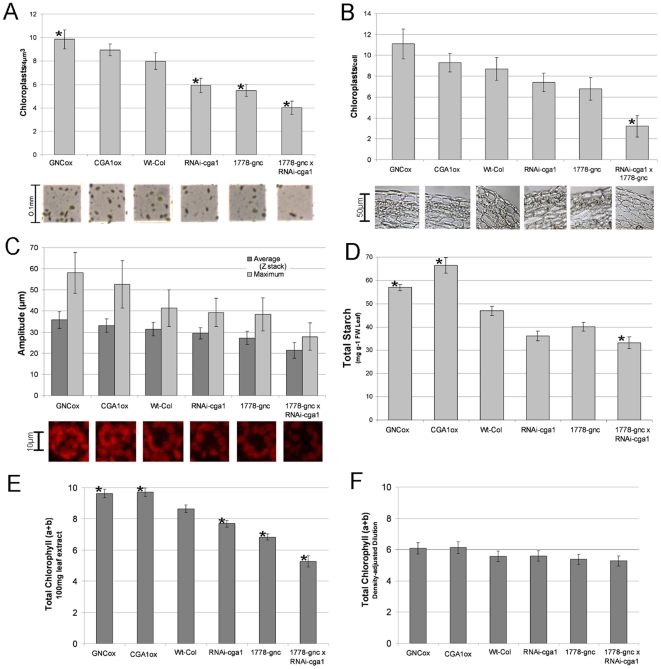
Chloroplast number and leaf starch in *GNC* and *CGA1* transgenics. A) Quantification of chloroplast numbers using hemocytometer following extraction from 100mg of rosette leaf tissue. B) Number of chloroplasts in chloroplast containing cells of the inflorescence from mature plants. Values are an average of counts from consecutive wax embedded sections taken from multiple plants from each line (n>3). C) Confocal microscopy of leaf tissue measuring auto-fluorescence of chlorophyll molecule. Images show obvious differences in chloroplast number between with changes in GNC and CGA1 expression. D) Starch measured from leaf material of 3 week old plants. Transgenic lines were compared to wild-type using the Megazyme Total Starch Assay kit. E) Chlorophyll quantified from chloroplasts extracted from 100mg leaf tissue using acetone based extraction. F) Chlorophyll quantified using acetone based technique following the application of a dilution factor to the extracted chloroplasts based on chloroplast counts. (All data are MEAN+SD, p<.05, t-test).

Cytokinin application has been shown to increase chloroplast numbers [63]. We questioned whether the differences in chlorophyll were specifically due to differences in chlorophyll biosynthesis in the *GNC* and C*GA1* transgenics or the result of altered chloroplast number. Chloroplasts were extracted from individual plants grown with sufficient nitrogen and quantified using a standard 0.1 mm hemocytometer. Differences in chloroplast number were comparable to chlorophyll measurements taken prior to extraction with overexpression lines showing 10–20% increases and all mutant lines showing reduced chloroplast number (Figure 6A). The double mutant retained less than 50% of the chloroplasts found in wild type plants (Figure 6A). These results were confirmed *in planta* using confocal microscopy and standard wax sectioning techniques. Counting chloroplasts from the outer sub-epidermal cells in consecutive stem sections demonstrated that the chloroplast number was significantly different (Figure 6B). Confocal microscopy on freshly harvested leaf tissue over a 30 µm depth revealed differences in chlorophyll auto-fluorescence as well as chloroplast number in mesophyll cells (Figure 6C). Chloroplasts are considered to be the primary site of starch biosynthesis [15]; thus, it is not surprising that leaf starch content was also found to be altered in cohort with chloroplast number (Figure 6D). Chlorophyll measurements were taken from extracted chloroplasts both before and after a dilution factor had been applied in order to correct for chloroplast number (Figure 6 E and F). Though there appear to be minor differences in chlorophyll content per chloroplast following dilution, these differences were not found to be significant. While these results indicate that there is increased overall chlorophyll biosynthesis, they also demonstrate that the difference in chlorophyll content in the GATA transgenic plants is primarily due to differences in chloroplast number (Figure 6E and F). Hence, expression of *GNC* and *CGA1* appear to modulate aspects of chloroplast development based on inputs from light and nitrogen, leading to differences in chlorophyll biosynthesis, chloroplast number and starch production.

### *GNC* and *CGA1* Modulate Expression of Chloroplast Localized Glutamate Synthase (*GLU1/Fd-GOGAT*) and Chlorophyll Biosynthesis Genes

**Figure 7:**
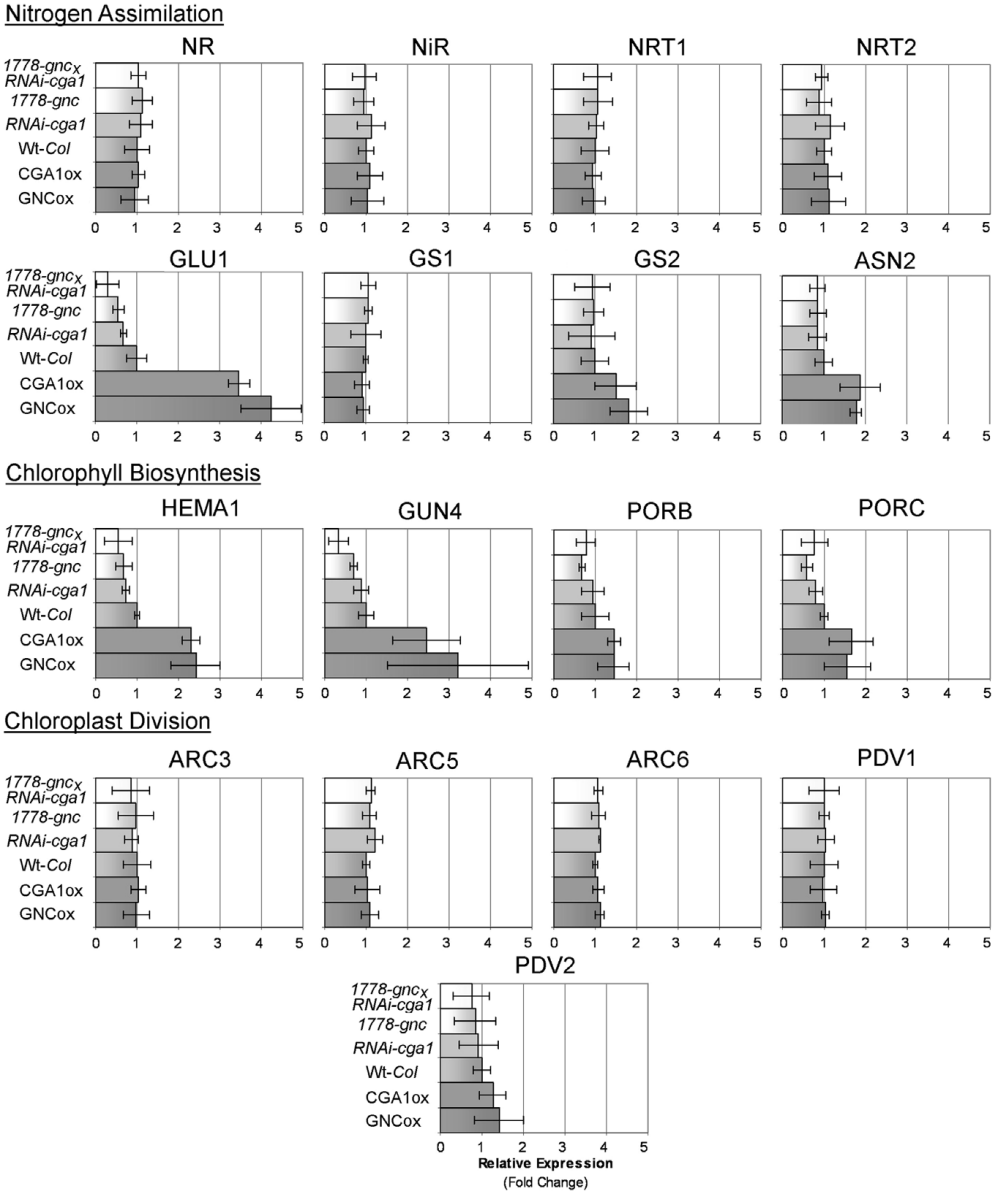
Quantitive Real-time RT-PCR analysis of gene expression from plants grown under sufficient nitrogen conditions. Relative expression of *GNC* and *CGA1* transgenic lines compared to Wt-*Col* under sufficient nitrogen conditions. Key genes involved in nitrogen assimilation, chlorophyll biosynthesis and chloroplast division were analyzed (At least 3 biological replicates). Genes involved in chloroplast-localized nitrogen assimilation and chlorophyll biosynthesis demonstrate correlation of expression with that of *GNC* and *CGA1*.

Following the uptake of inorganic nitrate from the soil, NO_3_^−^ is reduced to nitrite NO_2_^−^ by *NITRATE REDUCTASE* (NR) in the cytosol [64] Nitrite subsequently enter the chloroplast where *NITRITE REDUCT*ASE (*NiR*) converts nitrite to ammonium (NH4+) [64]. Through the GS/GOGAT cycle, ammonium is then incorporated into glutamate, which feeds directly into both amino acid synthesis and the C_5_ chlorophyll biosynthesis pathway [65]. Because nitrogen is a strong modulator of both *GNC* and *CGA1* expression [42] as well as chloroplast development, we analyzed the expression of some key nitrogen assimilation genes. Genes involved in preliminary nitrogen reduction in the cytosol (*NR, NiR*) and nitrogen transport (*NRT1, NRT2*) were not found to be altered in the *GNC* and *CGA1* transgenic lines. In plants grown at sufficient nitrogen conditions, chloroplast localized ferredoxin-dependent *GLUTAMATE SYNTHASE* (*GLU1/Fd-GOGAT*) expression was significantly increased by *GNC* and *CGA1* overexpression and decreased in the *gnc*, *RNAi-cga1* and double mutant lines (Figure 7). Mutation of *GLU1* drastically influences chloroplast development, leading to chlorotic or lethal plants under photorespiratory conditions [14], [66], [67]. While chloroplast localized *GLUTAMINE SYNTHETASE* (*GS2*) and *ASPARAGINE SYNTHASE* (*ASN*2) also show differences in expression, these were minor in comparison to *GLU1* (Figure 7). Expression of these genes has been shown to be altered with changes in *GLU1* [14]. Changes in gene expression from plants grown with high light and excess nitrogen (Figure S1) were found to be less significant in comparison to from the differences observed from plants grown with only sufficient nitrogen levels.

**Figure 8:**
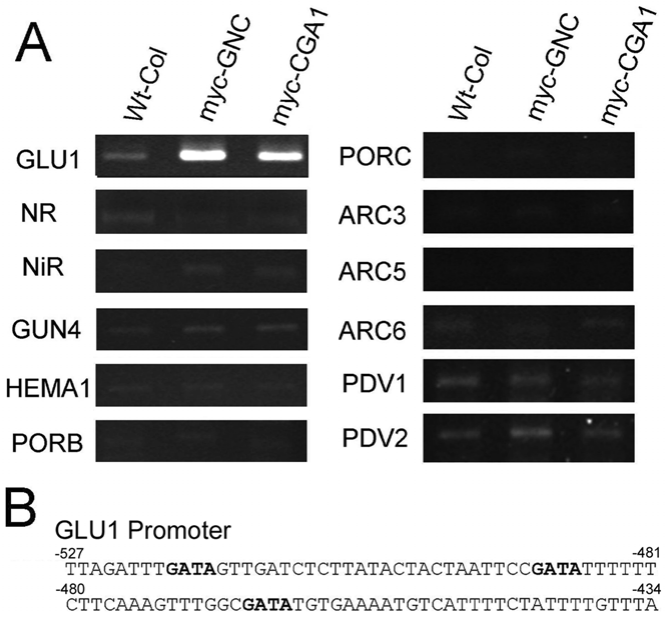
Chromatin immunnoprecipitation (ChIP) of myc-tagged GNC and CGA1 protein indicates interaction with the *GLU1* promoter. A) PCR of promoter regions containing GATA sites from suspected candidate genes following precipation with c-myc antibody using extracts from wild type plants and transgenics lines expressing myc-tagged *GNC* or *CGA1*. B) Region of the *GLU* promoter containing mutiple GATA sites suspected to be binding sites for GNC and CGA1 proteins.

Richter et al. [38], recently reported differences in *POR* gene expression in transgenic lines with altered *GNC* and *CGA1*. We analyzed the expression of these genes as well as expression of the key rate-limiting enzymes *HEMA1* and *GUN4*, which are found upstream in the chlorophyll biosynthetic pathway. These important chlorophyll biosynthesis genes were also found to be modified in correlation with expression levels of *GNC* and/or *CGA1* (Figure 8). *GUN4* and *HEMA1* display overlapping spatial and temporal expression with *GNC* and *CGA1* and also exhibit nearly identical circadian oscillations, resulting in a strong level of co-expression [68], [69]. These results validate the systems biology approach that predicted *GNC* and *CGA1* act as part of a network with key chlorophyll biosynthetic genes, specifically *GUN4* [50]. As seen with *GLU1*, *GNC* and *CGA1*, altering the expression of *GUN4* also results in altered chlorophyll biosynthesis [19]. *GUN4* has been shown to sustain chlorophyll levels under fluctuating environmental conditions and has been suggested to be involved in retrograde signaling to the nucleus, regulating *PORB*, *PORC* and chlorophyll-binding light harvesting complex (*LHC*) genes [19]. Because *GNC* and *CGA1* modulate the expression of *GLU1* and *GUN4*, it is likely that changes in gene expression found with changes in their expression will also be altered in *GNC* and *CGA1* transgenics. As such, carbon metabolism-related genes found to be significantly different *GNC* and *CGA1* transgenics [42], [44] may be indirectly modified as a consequence of altered chlorophyll biosynthesis.

Changes in chloroplast numbers indicated that genes involved in chloroplast division might also be altered in the GATA lines. However, we did not find significant changes in the expression of the chloroplast division genes analyzed which includes *PDV* genes *(PLASTID DIVISION)* and *ARC* genes *(ACCUMULATION AND REPLICATION OF CHLOROPLASTS*) (Figure 7). There are potentially small changes in the transcript levels of *PDV2*; however, these are not statistically significant. Altering expression of *PDV* and *ARC* genes has a drastic effect on chloroplast development resulting in a significant trade-off between total chloroplast number and chloroplast size and/or function [70]–[72]. As such, even slight manipulation of their expression may account for the moderate differences in chloroplast number (10–20% increase) observed in with increased expression of *GNC* and *CGA1*. However, these plastid division genes analyzed are of cyanobacterial ancestry [6] and do not show circadian oscillations or strong co-expression with *GNC* and *CGA1* [68], [69]. Thus, it is not surprising that their expression is not significantly modulated in the *GNC* and *CGA1* transgenic lines. While these genes represent some of the basic division machinery in *Arabidopsis*, there are a number of other genes that have been shown to alter chloroplast number which may also display differential activity with changes in *GNC* and *CGA1* expression [6], [73]. Furthermore, altering gene expression may not be a prerequisite for the plastid division process, which may be controlled at the level of protein activity. Using the *Arabidopsis* Co-expression Tool (ACT), analysis of either *GNC* with *GLU1* or *CGA1* with *GLU1* indicates that a number of genes potentially involved in the chloroplast division and starch synthesis are similarly expressed [68]. These include FtsH proteases (At1g50250, At5g58870, At5g42270) known to be involved in plastid division [74], [75]. Further analysis will aim to identify exactly how *GNC* and *CGA1* influence of the gene expression or activity of genes involved in chloroplast division processes.

### Chromatin Immunoprecipitation confirms *GLU1/Fd-GOGAT* as a Potential Target of *GNC* and *CGA1*

Being GATA transcription factors, *GNC* and *CGA1* are presumed to control the transcription of genes by binding directly to GATA sites within their promoter. GATA sites are prominent in the promoter regions of many of the genes that were predicted to be regulated by *GNC* and *CGA1*. Chromatin immunoprecipitation (ChIP) experiments were performed using extracts from transgenic lines expressing myc-tagged *GNC* or *CGA1* protein. A region of the promoter from *GLU1/Fd-GOGAT* was found to positively interact with both myc-*GNC* and myc-*CGA1* protein following immunoprecipitation (Figure 8). This region of the *GLU1* promoter contains multiple GATA sites that are likely important for interaction with *GNC* and *CGA1* (Figure 8). Promoter regions from the other nitrogen assimilation, chlorophyll biosynthesis and chloroplast division genes did not appear to show positive interaction with the myc-tagged *GNC* and *CGA1* protein. Though this does not rule other genes out as candidates for being directly regulated by *GNC* and *CGA1*, it does indicate that these transcription factors positively control nitrogen assimilation in the chloroplast through regulation of *GLU1*.

## Discussion

Control of chlorophyll biosynthesis and chloroplast development is vital for plants to optimize photosynthetic capture while maintaining the carbon∶nitrogen balance. By increasing the expression of *GLU1/Fd-GOGAT* as well as key chlorophyll biosynthesis genes, *GNC* and *CGA1* act to increase the flux of assimilated nitrogen towards chlorophyll production. GLU1 accounts for more than 96% of the total GOGAT activity in photosynthetic green leaves and has been verified as the primary nitrogen assimilation enzyme [67]. Altering *GLU1* expression has also been shown to result in changes to amino acid production and lead to a cascade of changes gene expression that subsequently influence many aspects of plant development [12], [14]. GLU1 plays a significant role in photorespiration, re-assimilating ammonium produced through this process [67]. Growth in non-photorespiratory conditions (high CO_2_) recovers the reduced chlorophyll of *glu1* mutants [67]. The amount of ammonium released by photorespiration is up to 10 times the amount of nitrogen taken up by the plant [76]. Therefore, the photorespiration nitrogen cycle and specifically regulation of *GLU1* is important for maintaining nitrogen assimilation and carbon balance [77].

Multiple studies have implicated GATA motifs in regulating important nitrogen genes as well as light signaling elements. The importance of GATA sites in controlling the initial stages in nitrogen uptake and nitrogen catabolism in fungi is well documented [78]–[81]. In addition, GATA motifs were previously suggested to regulate *GLU1* expression [66], [82], [83]. While *GNC* and *CGA1* do not appear to regulate nitrogen-related processes upstream of the chloroplast (uptake and reduction), modulation of *GLU1* provides a way to increase re-assimilation of photorespiratory products specifically within the chloroplast. Both the ammonia coming from primary nitrogen assimilation and the ammonia released by photorespiration appear to converge into the same pathway [77]. As such, increasing the amount of supplied nitrogen will ultimately decrease the demand for re-assimilating photorespiratory products, thus potentially masking the effects of altered *GNC* and *CGA1* expression (Figure 5). Regardless of the source of nitrogen entering the GS/GOGAT cycle, *GNC* and *CGA1* appear to increase the flux of assimilated nitrogen towards chlorophyll biosynthesis.

Cytokinin application has been shown to increase chlorophyll levels, chloroplast numbers, protein, sugar and starch concentrations while also inhibiting nitrogen remobilization [31], [63]. Increased chlorophyll concentration and chloroplast number may permit increased photosynthetic capture when conditions are optimal. Sugar levels, light and nitrogen are known to increase *GLU1* expression, reflecting the intricate balance between carbon and nitrogen metabolism [67], [84]. Increased carbon fixation will ultimately result in more carbon skeletons (sugars) to be used for energy production, stored as starch, or diverted through the TCA cycle for incorporation into glutamate [65]. Differences in photorespiration rates for plants treated with nitrate or ammonium are directly related to the production of 2-oxoglutarate (2-OG) and photorespiratory refixation [77], [85]. Though *GNC* and *CGA1* do not appear to directly regulate sugar sensing and signaling (Figure 1), genes involved in photosynthesis and carbon-metabolism may be altered indirectly as a result of increased chlorophyll concentration and chloroplast number.

The circadian clock at least in part regulates the expression of *GNC* and *CGA1* and the circadian control genes *CIRCADIAN CLOCK-ASSOCIATED 1* (*CCA1*) and *LATE ELONGATED HYPOCOTYL* (*LHY*) show strong co-expression with *GNC* and *CGA1* [7], [46], [86]. The expression of *GLU1*, *GUN4* and *HEMA1* are also under similar circadian control [15], [22], [87]and each of these are expressed at a significant level to support chlorophyll biosynthesis in the absence of *GNC* and *CGA1* [38], [44]. Thus, the presence of the GATA motif in the *GLU1* promoter is not required for expression. Instead, regulating the expression of *GNC* and *CGA1* appears to allow for modulation of chlorophyll biosynthesis. Though light is intrinsically linked to the circadian clock and establishes the initial circadian cycle in plants, evidence indicates nitrate, cytokinin, and GA signaling all receive input from the circadian clock as well [88]–[93]. The absence of an effect of GA on *NR* and *NiR* activities as well as nitrogen content indicates that like *GNC* and *CGA1*, GA does not play a role in controlling the preliminary stages of nitrate assimilation [94]. However, the finding that PIF3 binds upstream of the coding regions of *GNC* and *CGA1* and reduces their expression provides a direct link between GA signaling and that of light, nitrogen, cytokinin and the circadian clock [38].

PIF's demonstrate opposite expression to *GNC* and *CGA1*, and instead show co-expression with the key circadian oscillator *TOC1* (*TIMING OF CAB EXPRESSION 1)* that functions to positively control the level of *LHY/CCA1* [46], [95]. PIF's integrate the circadian clock, but do not play a significant role in controlling light input or function of the circadian clock [22], [88]. Instead, they negatively regulate chloroplast development specifically through repression of chloroplast biosynthesis and carotenoid genes including *GUN4*, *HEMA1* and *POR's* [22], [96]–[100]. Previously, PIF control over chloroplast development has primarily focused on the regulation of genes involved in GA signaling and/or directly on chlorophyll biosynthesis genes [51], [96]–[98], [101], [102]. Repression of *GNC* and *CGA1* provides evidence that PIF's also act at the level of chloroplast nitrogen assimilation. Differences in the expression of genes involved in nitrogen assimilation, including *GOGAT*, *GS*, and *ASN2*, have previously been reported in PIF transgenic lines [51], [100], [101]. Events downstream of nitrogen assimilation, chlorophyll biosynthesis and chloroplast development likely result in further differences in gene expression, such as the reported feedback regulation on GA-related genes [38]. Carbon availability has recently been linked to PIF signaling, potentially leading to further feedback loops regulating gene expression [103]. The carbon status and starch accumulation in particular may also exert influence over the chloroplast division process, leading to altered chloroplast numbers with changes to *GNC* and *CGA1*. While these relationships are not fully understood, current evidence suggests that light, nitrogen, and cytokinin signaling function to increase clock-regulated chloroplast biosynthesis by increasing the expression of *GNC* and *CGA1*, whereas PIF repression of their expression leads to the opposite effect.

Gene duplication can lead to divergence into distinct biological roles or may simply reflect a degree of functional redundancy [42]. From sequence alone, partial divergence of these paralogs seems likely as *CGA1* is a shorter gene with significant variation outside of the GATA zinc finger region [82]. Though *GNC* and *CGA1* have partially redundant roles in controlling chloroplast development, our results indicate they have partially divergent functions, with *CGA1* exhibiting a more direct influence over cytokinin and GA-related developmental processes. As predicted by co-expression analysis [46], [68], we found that *GNC* had a more significant effect on chloroplast development, while *CGA1* plays a more prominent role in maintaining the balance between cytokinin and gibberellins signaling Unlike Richter et al. [38], we did not observe significant differences in germination or the timing of developmental events in *GNC* transgenic lines. Previous studies have also failed to report a significant difference in the flowering time of the *gnc* mutant [38], [42], [44]. If *GNC* exerts control over this process, we would expect to see reciprocal differences between the mutant and over-expression lines as seen for CGA1. We speculate that this discrepancy in flowering time reported for *GNC* over-expression lines may be due to minor variations in growth conditions, such as light and nitrogen levels. It is also possible that different levels of overexpression may result in variations in phenotype, with higher than our observed 4-fold increase causing a more significant effect. Just as increased concentrations of cytokinin result in a more significant influence on plant growth, differences in the level of expression of either *GNC* or *CGA1* may exacerbate phenotypes related to cytokinin and GA signaling. Still, results suggest that *GNC* and *CGA1* have at least partially redundant roles and act at an important hub where light, nitrogen, cytokinin and GA signaling all converge.

Modification of both gibberellin [39], [104] and cytokinin [33], [105] signaling pathways has been used to increase agricultural productivity. Cross-talk between GA and cytokinin may involve differential regulation of transcription factors by these hormone signaling pathways. The control of *GNC* and *CGA1* expression appears to represent a pivotal point in the cross-talk between cytokinin and GA for regulating chloroplast development, possibly by influencing the amount of nitrogen being assimilated. Understanding how plants regulate chloroplast development has important implications for agricultural biotechnology. Crop plants require copious amounts of applied nitrogen fertilizer and crop yield is typically proportional to the amount of nitrogen available in the soil [1], [106]. Field conditions are not always optimal and crops typically experience periods of decreased light, water and nutrients. Uneven application of fertilizer and leeching of nitrogen from the soil also contributes to reduced yields [26]. Being able to maintain chlorophyll levels even when light and nitrogen are not abundant could be beneficial to crop plants. The conservation of the GATA family between *Arabidopsis* and rice [82] indicates that orthologs for *GNC* and *CGA1* genes perform similar roles in crop plants. Modulation of their expression could potentially allow for increased productivity under reduced nitrogen fertilizer conditions. Application of nitrogen fertilizer is costly both environmentally and economically; therefore any improvement that leads to increased nitrogen use efficiency could have a significant impact.

## Methods

### Growth conditions

*Arabidopsis* was typically grown in standard long-day (LD) conditions with 16 h of white light (150 µmol/m^2^-s) at 23°C and 8 h of darkness at 21°C. Short day (SD) conditions had reversed 8∶16 light cycle. We used nutrient-free soil Sunshine Mix #2 Basic (SunGro Horticulture Canada Ltd), adding nutrient solution with different amounts of nitrate as previously described [23], [107] with 10 mM nitrate solution as ‘sufficient’ for *Arabidopsis* growth, whereas 3 mM nitrate is a ‘limiting’ nitrogen condition, under which effects of nitrogen stress can be observed . ‘Full nutrient’ conditions consisted of a high nitrogen treatment with fertilized water (18-9-18) containing nitrogen @ 200 ppm and chelated micronutrients (PlantsProducts, #11072) and double the light intensity (300 µmol/m^2^-s).

For expression studies, plants were grown according to Harmer et al. [7], using a 12∶12 day night cycle. For germination, standard 1× MS salts were used and media supplemented with 1% Sucrose (Standard), 6% glucose (High Sugar) or 3 uM GA_3_ (Gibberellin).

### Transgenic *Arabidopsis* lines

Promoters from *GNC* and *CGA1* (∼1500 bp) were cloned into the pCambia3301 vector. The resultant *pGNC∶GUS* and *pCGA1∶GUS* constructs were amplified in *E. coli* DH10b cells and transformed into *Agrobacterium* strain EHA105. Wild type (Wt-*Col*) plants were transformed with either *pGNC∶GUS*-3301 or *pCGA1∶GUS*-3301 via the standard floral dip method [108] and screened with the herbicide BASTA (Hoechst). Tissue from homozygous lines was stained for GUS activity according to standard protocols.

35S∶*GNC* overexpression lines and the SALK01778-*gnc* mutant used in comparative analysis came from our previous work [42]. The *HXK1* mutant was the *gin2-1* line [53], [109] and the cytokinin receptor mutant used was the *ahk2-5/ahk3-7* line.

To suppress expression of *CGA1*, dsRNAi constructs were created the standard Gateway protocol (Invitrogen; Gateway Technology Manual, 2003). The dsRNAi insert contains a unique inverted repeat cDNA of *CGA1* (sequence positions 52–509 on CDS) separated by a backbone sequence. Each insert was placed behind a synthetic promoter derived from a maize *UBQ* promoter sequence and built into a SpecR binary construct containing the *PMI (Phosphomannose Isomerase*) selectable marker under the control of an *Actin* promoter. The resulting vector was transferred to *Agrobacterium tumefaciens* strain LBA4404, transformed using floral dip and transformants screened with BASTA (Hoechst). A modified pEGAD vector was used to overexpress *CGA1*. The 35S∶CaMV promoter was replaced with the *AtUBQ3* promoter. *CGA1* was amplified from wild type cDNA by RT-PCR and cloned into the modified pEGAD vector. This was amplified in *E. coli* DH10b cells, purified, transferred into *Agrobacterium* cell EHA105 and transformed using floral dip [108] and transformants selected with BASTA (Hoechst).

To generate c-myc tagged proteins, the cDNA sequences of *GNC* and *CGA1* were amplified from wild type cDNA and cloned into pGBKT7 vector (Clontech) next to the MYC sequence. To construct the binary vector, plasmid DNA was purified from *E. coli* DH10b cells using a DNA Maxiprep kit (Qiagen) and the *myc*-cDNA were amplified by PCR and cloned into the standard 35S pEGAD expression vector, minus the EGFP. This construct was amplified in *E. coli* and then transformed to *Agrobacterium* strain EHA105. *Wt*- Col plants were transformed with P35S-*myc-GNC*-pEGAD or P35S-*myc-CGA1*-pEGAD by the floral dip [108] and selected with BASTA (Hoechst). Primers used for genotyping are listed in Table S1 and those used in cloning procedures are outlined in Table S2.

### Chlorophyll and Chloroplast Measurements

Total chlorophyll levels were measured from the sixth leaf of plants of the same age using both the Minolta SPAD 502DL chlorophyll meter (Minolta) as well as the standard acetone extraction and spectrophotometeric quantification technique [110].

For measurements of leaf size, images of plant tissues were scaled and quantified using ImageJ Software (http://rsbweb.nih.gov/ij/).

Confocal microscopy was performed on fresh mounted leaf tissue using a Leica CM-1000microscope scanning auto-florescence of chlorophyll molecule and analysis was performed with LCS Lite software (Leica Microsystems Heidelberg GmbH) as previously described [83]. Wax embedding and sectioning was performed according to standard protocols using a Leica RM-2265 microtome and consecutive 12 µm sections from the same area of stem (5 cm from base) were photographed on light microscope using Qcapture software (Qimaging).

To collect intact chloroplast, *Arabidopsis* rosette leaves were cut into small squares (approx 0.5 cm2), rinsed with distilled water and put in 2 ml round-bottom tube with 1 ml of ice cold Chloroplast Extraction Buffer (50 mM Hepes buffer pH 8.0, 5 mM MgCl, 1 mM EDTA, 1 mM DTT, 30% sorbitol and protease inhibitors). A round, metallic grinding bead was added and tissue ground using a Retsch Mill (MM 200) at low speed with multiple reps of 1 minute until is tissues is completely broken up. Extracts were filtered through layer of Miracloth and 100 µm nylon mesh that was pre-soaked in ice-cold Extraction Buffer. This process was repeated and chloroplasts pooled following low speed centrifugation (3000 g). Chloroplast suspension were loaded onto preformed Percoll gradient according to manufactures instructions (GE Healthcare Life Sciences) and centrifuged at 5000 g for 10 minutes at 4°C. Intact chloroplasts were collected and re-suspended 1 ml of Extraction Buffer. Extracts were again centrifuged for 10 minutes at 3000 g and the final pellet re-suspended for quantification using 0.1 mm hemocytometer or acetone for chlorophyll extraction. All statistical analyses were performed with JMP Statistical Analysis Software (www.jmp.com).

### Starch Analysis

For Starch quantification, 100 mg fresh weight of mature fully expanded *Arabidopsis* leaves were flash frozen in liquid nitrogen, ground to a fine powder and extracted with 1 mL 100% methanol by shaking at 70°C for 15 min. The extraction was repeated two times and insoluble residue was freeze dried overnight, weighed, and starch content was determined with the Amylase/Amyloglucosidase method using the Megazyme Total Starch Assay kit according to the manufacturer's instructions (Megazyme International Ireland Ltd.)

### Semi-quantitative RT-PCR and quantitative Real-time PCR

Total RNA was extracted from 100 mg leaf tissue using Trizol (Invitrogen), treated with DNAse (Promega) and purified using RNeasy Mini kit (Qiagen). Extracts were quantified using the Nanodrop ND-1000 (Nanadrop) and first strand synthesis of cDNA was performed using qScript cDNA SuperMix (Quanta Biosciences) from 1 µg total RNA. For semi-quantitative RT-PCR, reactions were performed using GoTaq Flexi (Promega) and expression of transgenic lines was quantified using ImageJ Software. *Ubiquitin* (*UBQ10*) was used as endogenous control.

Quantitative real-time expression was performed using PerfeCTa SYBR Green SuperMix ROX (Quanta Biosciences) on the ABI7300 (Applied Biosystems). Primers were selected from previous publications or designed using the Applied Biosystems software PRIMER EXPRESS 2.0. The corresponding 7300 system software 1.2.2 and the Applied Biosystems relative quantification study software 1.2.2 (Applied Biosystems) was used for analysis of expression levels with *GAPDH* as endogenous control. Primers used in expression analysis are listed in Table S3.

It should be noted that analyzing the expression of genes exhibiting circadian oscillations requires specific care to be taken with respect to sampling time. Because the baseline expression level of *GNC* and *CGA1* are in constant circadian flux, differences in gene expression were found to be dependent on the time of day that the experiment was performed. Samples taken from different times of day also produce high levels of variation when compared. For this reason, comparisons of gene expression were made from samples harvested when endogenous levels of both *GNC/CGA1* and *HEMA1/GUN4* are lowest [46].

### Chromatin Immunoprecipitation

ChIP experiments were perform on the myc-tagged plants lines with the EZ ChIP™ Chromatin Immunoprecipitation (Millipore) kit according to manufacturers instructions using the monoclonal anti c-myc-antibody (Sigma). Following immunoprecipitation standard PCR reactions were performed using primers directed against promoter regions of suspected target genes containing GATA sites (Table S4) . PCR reactions were performed on both direct extract (input) and no-antibody controls to ensure specificity of c-myc antibody. For each extract, RNA polymerase II provided with kit was used as internal standard.

## Supporting Information

Figure S1 **Quantitive Real-time RT-PCR analysis of gene expression from plants grown under full nutrient conditions.** Relative expression of GATA lines compared to Wt-*Col* grown with 300 µmol/m^2^-s light and full nutrient fertilizer. Samples were taken from 100 mg of rosette leaf of 3 week old plants. Key genes involved in nitrogen assimilation, chlorophyll biosynthesis and chloroplast division analyzed (At least 3 biological replicates). Similar to chlorophyll readings, differences in gene expression are not as large under increased nutrient conditions as those taken from plant grown with reduced nutrients.
(TIF)

Table S1 PCR primers used in genotyping *gnc* mutants and RNAi-*cga1* lines.
(DOC)

Table S2 PCR primers used in cloning for *UBQ∶CGA1* over-expression and *35S∶myc*-tagged lines.
(DOC)

Table S3 Primers used for semi-quantitative RT- PCR and quantitative Real-time (qRT) PCR analysis of gene expression.
(DOC)

Table S4 PCR primers used for ChIP analysis of myc-tagged GNC and CGA1.
(DOC)

## References

[1] Rothstein SJ (2007). Returning to our roots: Making plant biology research relevant to future challenges in agriculture.. Plant Cell.

[2] Hirel B, Le Gouis J, Ney B, Gallais A (2007). The challenge of improving nitrogen use efficiency in crop plants: Towards a more central role for genetic variability and quantitative genetics within integrated approaches.. J Exp Bot.

[3] Forde BG (2002). Local and long-range signalling pathways regulating plant responses to nitrate.. Annual Review of Plant Biology Vol.

[4] Lopez-Juez E, Pyke KA (2005). Plastids unleashed: Their development and their integration in plant development.. Int J Dev Biol.

[5] Barry CS (2009). The stay-green revolution: Recent progress in deciphering the mechanisms of chlorophyll degradation in higher plants.. Plant Science.

[6] Okazaki K, Kabeya Y, Miyagishima SY (2010). The evolution of the regulatory mechanism of chloroplast division.. Plant Signal Behav.

[7] Harmer SL, Hogenesch JB, Straume M, Chang HS, Han B (2000). Orchestrated transcription of key pathways in *Arabidopsis* by the circadian clock.. Science.

[8] McClung CR (2006). Plant circadian rhythms.. Plant Cell.

[9] Nozue K, Covington MF, Duek PD, Lorrain S, Fankhauser C (2007). Rhythmic growth explained by coincidence between internal and external cues.. Nature.

[10] Coruzzi G, Bush DR (2001). Nitrogen and carbon nutrient and metabolite signaling in plants.. Plant Physiol.

[11] Coruzzi GM, Zhou L (2001). Carbon and nitrogen sensing and signaling in plants: Emerging ‘matrix effects’.. Curr Opin Plant Biol.

[12] Ishizaki T, Ohsumi C, Totsuka K, Igarashi D (2009). Analysis of glutamate homeostasis by overexpression of Fd-GOGAT gene in *Arabidopsis thaliana*.. Amino Acids.

[13] Potel F, Valadier MH, Ferrario-Mery S, Grandjean O, Morin H (2009). Assimilation of excess ammonium into amino acids and nitrogen translocation in *Arabidopsis thaliana*–roles of glutamate synthases and carbamoylphosphate synthetase in leaves.. FEBS J.

[14] Kissen R, Winge P, Tran DH, Jorstad TS, Storseth TR (2010). Transcriptional profiling of an Fd-GOGAT1/GLU1 mutant in *Arabidopsis thaliana* reveals a multiple stress response and extensive reprogramming of the transcriptome.. BMC Genomics.

[15] Eckhardt U, Grimm B, Hortensteiner S (2004). Recent advances in chlorophyll biosynthesis and breakdown in higher plants.. Plant Mol Biol.

[16] Kumar AM, Soll D (2000). Antisense HEMA1 RNA expression inhibits heme and chlorophyll biosynthesis in *Arabidopsis*.. Plant Physiol.

[17] Hedtke B, Alawady A, Chen S, Bornke F, Grimm B (2007). HEMA RNAi silencing reveals a control mechanism of ALA biosynthesis on mg chelatase and fe chelatase.. Plant Mol Biol.

[18] Adhikari ND, Orler R, Chory J, Froehlich JE, Larkin RM (2009). Porphyrins promote the association of GENOMES UNCOUPLED 4 and a mg-chelatase subunit with chloroplast membranes.. J Biol Chem.

[19] Peter E, Grimm B (2009). GUN4 is required for posttranslational control of plant tetrapyrrole biosynthesis.. Mol Plant.

[20] Davison PA, Schubert HL, Reid JD, Iorg CD, Heroux A (2005). Structural and biochemical characterization of Gun4 suggests a mechanism for its role in chlorophyll biosynthesis.. Biochemistry.

[21] Paddock TN, Mason ME, Lima DF, Armstrong GA (2010). *Arabidopsis* protochlorophyllide oxidoreductase A (PORA) restores bulk chlorophyll synthesis and normal development to a porB porC double mutant.. Plant Mol Biol.

[22] Stephenson PG, Fankhauser C, Terry MJ (2009). PIF3 is a repressor of chloroplast development.. Proc Natl Acad Sci U S A.

[23] Peng M, Bi YM, Zhu T, Rothstein SJ (2007). Genome-wide analysis of *Arabidopsis* responsive transcriptome to nitrogen limitation and its regulation by the ubiquitin ligase gene NLA.. Plant Mol Biol.

[24] Bondada BR, Syvertsen JP (2003). Leaf chlorophyll, net gas exchange and chloroplast ultrastructure in citrus leaves of different nitrogen status.. Tree Physiol.

[25] Hussain F, Bronson KF, Yadvinder-Singh, Bijay-Singh, Peng S (2000). Use of chlorophyll meter sufficiency indices for nitrogen management of irrigated rice in asia.. Agron J.

[26] Scharf PC, Brouder SM, Hoeft RG (2006). Chlorophyll meter readings can predict nitrogen need and yield response of corn in the north-central USA.. Agron J.

[27] Takei K, Sakakibara H, Taniguchi M, Sugiyama T (2001). Nitrogen-dependent accumulation of cytokinins in root and the translocation to leaf: Implication of cytokinin species that induces gene expression of maize response regulator.. Plant Cell Physiol.

[28] Takei K, Takahashi T, Sugiyama T, Yamaya T, Sakakibara H (2002). Multiple routes communicating nitrogen availability from roots to shoots: A signal transduction pathway mediated by cytokinin.. J Exp Bot.

[29] Sakakibara H, Takei K, Hirose N (2006). Interactions between nitrogen and cytokinin in the regulation of metabolism and development.. Trends Plant Sci.

[30] To JP, Kieber JJ (2008). Cytokinin signaling: Two-components and more.. Trends Plant Sci.

[31] Argueso CT, Raines T, Kieber JJ (2010). Cytokinin signaling and transcriptional networks.. Curr Opin Plant Biol.

[32] Muller B, Sheen J (2007). *Arabidopsis* cytokinin signaling pathway.. Sci STKE.

[33] Ashikari M, Sakakibara H, Lin S, Yamamoto T, Takashi T (2005). Cytokinin oxidase regulates rice grain production.. Science.

[34] Bartrina I, Otto E, Strnad M, Werner T, Schmulling T (2011). Cytokinin regulates the activity of reproductive meristems, flower organ size, ovule formation, and thus seed yield in *Arabidopsis thaliana*.. Plant Cell.

[35] Greenboim-Wainberg Y, Maymon I, Borochov R, Alvarez J, Olszewski N (2005). Cross talk between gibberellin and cytokinin: The *Arabidopsis* GA response inhibitor SPINDLY plays a positive role in cytokinin signaling.. Plant Cell.

[36] Weiss D, Ori N (2007). Mechanisms of cross talk between gibberellin and other hormones.. Plant Physiol.

[37] Gan Y, Liu C, Yu H, Broun P (2007). Integration of cytokinin and gibberellin signalling by *Arabidopsis* transcription factors GIS, ZFP8 and GIS2 in the regulation of epidermal cell fate.. Development.

[38] Richter R, Behringer C, Muller IK, Schwechheimer C (2010). The GATA-type transcription factors GNC and GNL/CGA1 repress gibberellin signaling downstream from DELLA proteins and PHYTOCHROME-INTERACTING FACTORS.. Genes Dev.

[39] Peng J, Richards DE, Hartley NM, Murphy GP, Devos KM (1999). ‘Green revolution’ genes encode mutant gibberellin response modulators.. Nature.

[40] Feng S, Martinez C, Gusmaroli G, Wang Y, Zhou J (2008). Coordinated regulation of *Arabidopsis thaliana* development by light and gibberellins.. Nature.

[41] Cheminant S, Wild M, Bouvier F, Pelletier S, Renou JP (2011). DELLAs regulate chlorophyll and carotenoid biosynthesis to prevent photooxidative damage during seedling deetiolation in *Arabidopsis*.. Plant Cell.

[42] Bi YM, Zhang Y, Signorelli T, Zhao R, Zhu T (2005). Genetic analysis of *Arabidopsis* GATA transcription factor gene family reveals a nitrate-inducible member important for chlorophyll synthesis and glucose sensitivity.. Plant J.

[43] Naito T, Kiba T, Koizumi N, Yamashino T, Mizuno T (2007). Characterization of a unique GATA family gene that responds to both light and cytokinin in *Arabidopsis thaliana*.. Biosci Biotechnol Biochem.

[44] Mara CD, Irish VF (2008). Two GATA transcription factors are downstream effectors of floral homeotic gene action in *Arabidopsis*.. Plant Physiol.

[45] Monte E, Tepperman JM, Al-Sady B, Kaczorowski KA, Alonso JM (2004). The phytochrome-interacting transcription factor, PIF3, acts early, selectively, and positively in light-induced chloroplast development.. Proc Natl Acad Sci U S A.

[46] Manfield IW, Devlin PF, Jen CH, Westhead DR, Gilmartin PM (2007). Conservation, convergence, and divergence of light-responsive, circadian-regulated, and tissue-specific expression patterns during evolution of the *Arabidopsis* GATA gene family.. Plant Physiol.

[47] Scheible WR, Morcuende R, Czechowski T, Fritz C, Osuna D (2004). Genome-wide reprogramming of primary and secondary metabolism, protein synthesis, cellular growth processes, and the regulatory infrastructure of *Arabidopsis* in response to nitrogen.. Plant Physiol.

[48] Brenner WG, Romanov GA, Kollmer I, Burkle L, Schmulling T (2005). Immediate-early and delayed cytokinin response genes of *Arabidopsis thaliana* identified by genome-wide expression profiling reveal novel cytokinin-sensitive processes and suggest cytokinin action through transcriptional cascades.. Plant J.

[49] Kiba T, Naitou T, Koizumi N, Yamashino T, Sakakibara H (2005). Combinatorial microarray analysis revealing *Arabidopsis* genes implicated in cytokinin responses through the his->Asp phosphorelay circuitry.. Plant Cell Physiol.

[50] Needham CJ, Manfield IW, Bulpitt AJ, Gilmartin PM, Westhead DR (2009). From gene expression to gene regulatory networks in *Arabidopsis thaliana*.. BMC Syst Biol.

[51] Leivar P, Tepperman JM, Monte E, Calderon RH, Liu TL (2009). Definition of early transcriptional circuitry involved in light-induced reversal of PIF-imposed repression of photomorphogenesis in young *Arabidopsis* seedlings.. Plant Cell.

[52] Jang JC, Leon P, Zhou L, Sheen J (1997). Hexokinase as a sugar sensor in higher plants.. Plant Cell.

[53] Moore B, Zhou L, Rolland F, Hall Q, Cheng WH (2003). Role of the *Arabidopsis* glucose sensor HXK1 in nutrient, light, and hormonal signaling.. Science.

[54] Rolland F, Sheen J (2005). Sugar sensing and signalling networks in plants.. Biochem Soc Trans.

[55] Winter D, Vinegar B, Nahal H, Ammar R, Wilson GV (2007). An “electronic fluorescent pictograph” browser for exploring and analyzing large-scale biological data sets.. PLoS ONE.

[56] Higuchi M, Pischke MS, Mahonen AP, Miyawaki K, Hashimoto Y (2004). In planta functions of the *Arabidopsis* cytokinin receptor family.. Proc Natl Acad Sci U S A.

[57] Riefler M, Novak O, Strnad M, Schmulling T (2006). *Arabidopsis* cytokinin receptor mutants reveal functions in shoot growth, leaf senescence, seed size, germination, root development, and cytokinin metabolism.. Plant Cell.

[58] Huang S, Raman AS, Ream JE, Fujiwara H, Cerny RE (1998). Overexpression of 20-oxidase confers a gibberellin-overproduction phenotype in *Arabidopsis*.. Plant Physiol.

[59] Peng J, Carol P, Richards DE, King KE, Cowling RJ (1997). The *Arabidopsis* GAI gene defines a signaling pathway that negatively regulates gibberellin responses.. Genes Dev.

[60] Koornneef M, Bentsink L, Hilhorst H (2002). Seed dormancy and germination.. Curr Opin Plant Biol.

[61] Kim YC, Nakajima M, Nakayama A, Yamaguchi I (2005). Contribution of gibberellins to the formation of *Arabidopsis* seed coat through starch degradation.. Plant Cell Physiol.

[62] Jacobsen SE, Olszewski NE (1993). Mutations at the SPINDLY locus of *Arabidopsis* alter gibberellin signal transduction.. Plant Cell.

[63] Criado MV, Caputo C, Roberts IN, Castro MA, Barneix AJ (2009). Cytokinin-induced changes of nitrogen remobilization and chloroplast ultrastructure in wheat (*triticum aestivum*).. J Plant Physiol.

[64] Crawford NM, Glass ADM (1998). Molecular and physiological aspects of nitrate uptake in plants.. Trends Plant Sci.

[65] Gough SP, Westergren T, Hansson M (2003). Chlorophyll biosynthesis in higher plants. regulatory aspects of 5-aminolevulinate formation.. Journal of Plant Biology.

[66] Feraud M, Masclaux-Daubresse C, Ferrario-Mery S, Pageau K, Lelandais M (2005). Expression of a ferredoxin-dependent glutamate synthase gene in mesophyll and vascular cells and functions of the enzyme in ammonium assimilation in nicotiana tabacum (L.).. Planta.

[67] Coschigano KT, Melo-Oliveira R, Lim J, Coruzzi GM (1998). *Arabidopsis* gls mutants and distinct fd-GOGAT genes. implications for photorespiration and primary nitrogen assimilation.. Plant Cell.

[68] Jen CH, Manfield IW, Michalopoulos I, Pinney JW, Willats WG (2006). The *Arabidopsis* co-expression tool (ACT): A WWW-based tool and database for microarray-based gene expression analysis.. Plant J.

[69] Toufighi K, Brady SM, Austin R, Ly E, Provart NJ (2005). The botany array resource: E-northerns, expression angling, and promoter analyses.. Plant J.

[70] Miyagishima SY, Froehlich JE, Osteryoung KW (2006). PDV1 and PDV2 mediate recruitment of the dynamin-related protein ARC5 to the plastid division site.. Plant Cell.

[71] Okazaki K, Kabeya Y, Suzuki K, Mori T, Ichikawa T (2009). The PLASTID DIVISION1 and 2 components of the chloroplast division machinery determine the rate of chloroplast division in land plant cell differentiation.. Plant Cell.

[72] Glynn JM, Froehlich JE, Osteryoung KW (2008). *Arabidopsis* ARC6 coordinates the division machineries of the inner and outer chloroplast membranes through interaction with PDV2 in the intermembrane space.. Plant Cell.

[73] Maple J, Moller SG (2007). Plastid division: Evolution, mechanism and complexity.. Ann Bot.

[74] Zaltsman A, Ori N, Adam Z (2005). Two types of FtsH protease subunits are required for chloroplast biogenesis and photosystem II repair in *Arabidopsis*.. Plant Cell.

[75] Liu X, Yu F, Rodermel S (2010). *Arabidopsis* chloroplast FtsH, var2 and suppressors of var2 leaf variegation: A review.. J Integr Plant Biol.

[76] Rachmilevitch S, Cousins AB, Bloom AJ (2004). Nitrate assimilation in plant shoots depends on photorespiration.. Proc Natl Acad Sci U S A.

[77] Nunes-Nesi A, Fernie AR, Stitt M (2010). Metabolic and signaling aspects underpinning the regulation of plant carbon nitrogen interactions.. Mol Plant.

[78] Scazzocchio C (2000). The fungal GATA factors.. Curr Opin Microbiol.

[79] Rastogi R, Bate NJ, Sivasankar S, Rothstein SJ (1997). Footprinting of the spinach nitrite reductase gene promoter reveals the preservation of nitrate regulatory elements between fungi and higher plants.. Plant Mol Biol.

[80] Cooper TG (2002). Transmitting the signal of excess nitrogen in *saccharomyces cerevisiae* from the tor proteins to the GATA factors: Connecting the dots.. FEMS Microbiol Rev.

[81] Tate JJ, Georis I, Dubois E, Cooper TG (2010). Distinct phosphatase requirements and GATA factor responses to nitrogen catabolite repression and rapamycin treatment in *saccharomyces cerevisiae*.. J Biol Chem.

[82] Reyes JC, Muro-Pastor MI, Florencio FJ (2004). The GATA family of transcription factors in *Arabidopsis* and rice.. Plant Physiol.

[83] Jonassen EM, Sevin DC, Lillo C (2009). The bZIP transcription factors HY5 and HYH are positive regulators of the main nitrate reductase gene in *Arabidopsis* leaves, NIA2, but negative regulators of the nitrate uptake gene NRT1.1.. J Plant Physiol.

[84] Lam HM, Coschigano KT, Oliveira IC, Melo-Oliveira R, Coruzzi GM (1996). The molecular-genetics of nitrogen assimilation into amino acids in higher plants.. Annu Rev Plant Physiol Plant Mol Biol.

[85] Guo S, Zhou Y, Gao Y, Li Y, Shen Q (2007). New insights into the nitrogen form effect on photosynthesis and photorespiration.. Pedosphere.

[86] Alabadi D, Yanovsky MJ, Mas P, Harmer SL, Kay SA (2002). Critical role for CCA1 and LHY in maintaining circadian rhythmicity in *Arabidopsis*.. Curr Biol.

[87] Mas P, Yanovsky MJ (2009). Time for circadian rhythms: Plants get synchronized.. Curr Opin Plant Biol.

[88] Arana MV, Marin-de la Rosa N, Maloof JN, Blazquez MA, Alabadi D (2011). Circadian oscillation of gibberellin signaling in *Arabidopsis*.. Proc Natl Acad Sci U S A.

[89] Robertson FC, Skeffington AW, Gardner MJ, Webb AA (2009). Interactions between circadian and hormonal signalling in plants.. Plant Mol Biol.

[90] Gutierrez RA, Stokes TL, Thum K, Xu X, Obertello M (2008). Systems approach identifies an organic nitrogen-responsive gene network that is regulated by the master clock control gene CCA1.. Proc Natl Acad Sci U S A.

[91] Ito S, Nakamichi N, Nakamura Y, Niwa Y, Kato T (2007). Genetic linkages between circadian clock-associated components and phytochrome-dependent red light signal transduction in *Arabidopsis thaliana*.. Plant Cell Physiol.

[92] Lillo C, Meyer C, Ruoff P (2001). The nitrate reductase circadian system. the central clock dogma contra multiple oscillatory feedback loops.. Plant Physiol.

[93] Hanano S, Domagalska MA, Nagy F, Davis SJ (2006). Multiple phytohormones influence distinct parameters of the plant circadian clock.. Genes Cells.

[94] Bouton S, Leydecker M, Meyer C, Truong H (2002). Role of gibberellins and of the RGA and GAI genes in controlling nitrate assimilation in *Arabidopsis thaliana*.. Plant Physiology and Biochemistry.

[95] Hayama R, Coupland G (2003). Shedding light on the circadian clock and the photoperiodic control of flowering.. Curr Opin Plant Biol.

[96] Leivar P, Quail PH (2011). PIFs: Pivotal components in a cellular signaling hub.. Trends Plant Sci.

[97] Huq E, Al-Sady B, Hudson M, Kim C, Apel K (2004). Phytochrome-interacting factor 1 is a critical bHLH regulator of chlorophyll biosynthesis.. Science.

[98] Moon J, Zhu L, Shen H, Huq E (2008). PIF1 directly and indirectly regulates chlorophyll biosynthesis to optimize the greening process in *Arabidopsis*.. Proc Natl Acad Sci U S A.

[99] Toledo-Ortiz G, Huq E, Rodriguez-Concepcion M (2010). Direct regulation of phytoene synthase gene expression and carotenoid biosynthesis by phytochrome-interacting factors.. Proc Natl Acad Sci U S A.

[100] Shin J, Kim K, Kang H, Zulfugarov IS, Bae G (2009). Phytochromes promote seedling light responses by inhibiting four negatively-acting phytochrome-interacting factors.. Proc Natl Acad Sci U S A.

[101] Oh E, Kang H, Yamaguchi S, Park J, Lee D (2009). Genome-wide analysis of genes targeted by PHYTOCHROME INTERACTING FACTOR 3-LIKE5 during seed germination in *Arabidopsis*.. Plant Cell.

[102] Oh E, Yamaguchi S, Hu J, Yusuke J, Jung B (2007). PIL5, a phytochrome-interacting bHLH protein, regulates gibberellin responsiveness by binding directly to the GAI and RGA promoters in *Arabidopsis* seeds.. Plant Cell.

[103] Stewart JL, Maloof JN, Nemhauser JL (2011). PIF genes mediate the effect of sucrose on seedling growth dynamics.. PLoS One.

[104] Sasaki A, Ashikari M, Ueguchi-Tanaka M, Itoh H, Nishimura A (2002). Green revolution: A mutant gibberellin-synthesis gene in rice.. Nature.

[105] Peleg Z, Reguera M, Tumimbang E, Walia H, Blumwald E (2011). Cytokinin-mediated source/sink modifications improve drought tolerance and increase grain yield in rice under water-stress.. Plant Biotechnol J.

[106] Edgerton MD (2009). Increasing crop productivity to meet global needs for feed, food, and fuel.. Plant Physiol.

[107] Peng M, Hudson D, Schofield A, Tsao R, Yang R (2008). Adaptation of *Arabidopsis* to nitrogen limitation involves induction of anthocyanin synthesis which is controlled by the NLA gene.. J Exp Bot.

[108] Clough SJ, Bent AF (1998). Floral dip: A simplified method for agrobacterium-mediated transformation of *Arabidopsis thaliana*.. Plant J.

[109] Moore B, Sheen J (1999). Plant sugar sensing and signaling - a complex reality.. Trends Plant Sci.

[110] Inskeep WP, Bloom PR (1985). Extinction coefficients of chlorophyll a and B in n,n-dimethylformamide and 80% acetone.. Plant Physiol.

