# Supplementary figures and images for: GNC and CGA1 Modulate Chlorophyll Biosynthesis and Glutamate Synthase (GLU1/Fd-GOGAT) Expression in Arabidopsis
Source: PLoS One. 2011 Nov 10;6(11):e26765. doi: 10.1371/journal.pone.0026765 (PMC3213100; doi:10.1371/journal.pone.0026765)

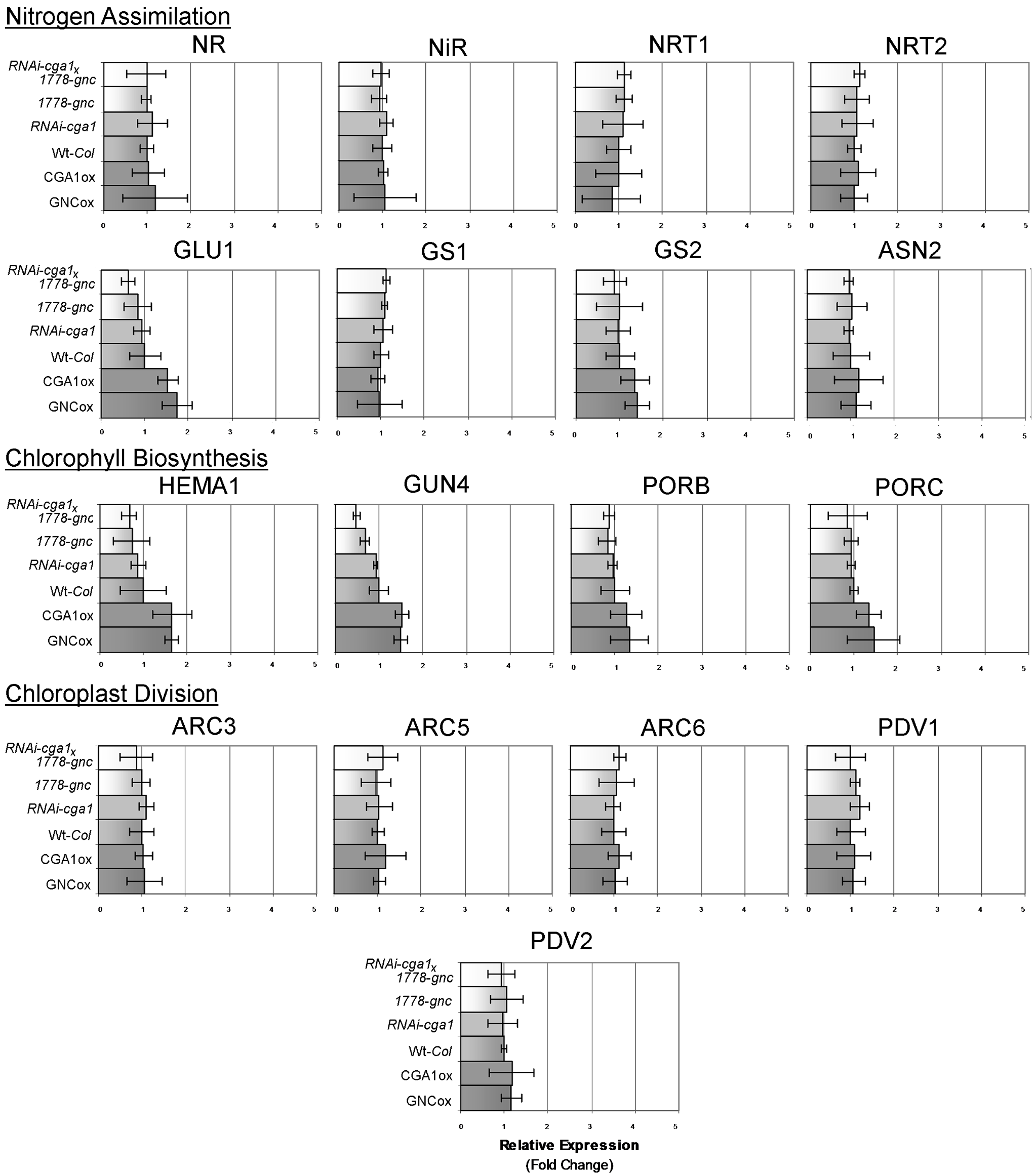

Supplement: Figure S1 — Quantitive Real-time RT-PCR analysis of gene expression from plants grown under full nutrient conditions. Relative expression of GATA lines compared to Wt-Col grown with 300 µmol/m2-s light and full nutrient fertilizer. Samples were taken from 100 mg of rosette leaf of 3 week old plants. Key genes involved in nitrogen assimilation, chlorophyll biosynthesis and chloroplast division analyzed (At least 3 biological replicates). Similar to chlorophyll readings, differences in gene expression are not as large under increased nutrient conditions as those taken from plant grown with reduced nutrients. (TIF) [file pone.0026765.s001.tif]
