# Supplementary material for: GNC and CGA1 Modulate Chlorophyll Biosynthesis and Glutamate Synthase (GLU1/Fd-GOGAT) Expression in Arabidopsis
Source: PLoS One. 2011 Nov 10;6(11):e26765. doi: 10.1371/journal.pone.0026765 (PMC3213100; doi:10.1371/journal.pone.0026765)
Supplement: Table S1 — PCR primers used in genotyping gnc mutants and RNAi-cga1 lines. (DOC) [file pone.0026765.s002.doc]

| **Table S1.** PCR primers used in genotyping *gnc* mutants and RNAi-*cga1* lines. F: forward primer; R: reverse primer. | |
| --- | --- |
| Primer Name | Sequence (5´ to 3´) |
| GNC | F: GCCACCATTGGAGGAGAGTGA  R: CAGGCCAAGATGTTTGTGGCT |
| SALK01778_ *gnc* insert | F: TGGTTCACGTAGTGGGCCATCG (Lba1)  R: CAGGCCAAGATGTTTGTGGCT |
| CGA1 | F: Tagaccggtatgggttccaattttcattac  R: caagaattcacccgtgaaccattccg |
| PMI | F: CGCCAGCCTGTTGAATATGC  R: ACGTTGCATCGCCTTCGAC |
