# Supplementary material for: GNC and CGA1 Modulate Chlorophyll Biosynthesis and Glutamate Synthase (GLU1/Fd-GOGAT) Expression in Arabidopsis
Source: PLoS One. 2011 Nov 10;6(11):e26765. doi: 10.1371/journal.pone.0026765 (PMC3213100; doi:10.1371/journal.pone.0026765)
Supplement: Table S2 — PCR primers used in cloning for UBQ∶CGA1 over-expression and 35S∶myc-tagged lines. (DOC) [file pone.0026765.s003.doc]

| **Table S2.** PCR primers used in cloning for *UBQ:CGA1* over-expression and *35S:myc-*tagged lines.  F: forward primer; R: reverse primer. | |
| --- | --- |
| Primer Name | Sequence (5´ to 3´) |
| UBQ3 | F: attcgagctccggtaccg  R: gctctagaccggtcaagatccacctgaaataaaac |
| CGA1 cDNA | F: Tagaccggtatgggttccaattttcattac R:caagaattcacccgtgaaccattccg |
| myc-GNCPpro | F: GCTATTCCATATGGATTCAAATTTTCATTACTCGATAG  R: CCGGAATTCACCGTGAACCATTCCATAC |
| myc-CGA1Ppro | F: GCTCTTGATGAATCACATGCGATAGAAAG  R: CATGGGACCGATAGAGATCGATGAAGC |
| myc-GNC cDNA | F:GGACTAGTATGGAGGAGCAGAAGCTGATC  R:CCGGAATTCTCAACCGTGAACCATTCCATAC |
| myc-CGA1 cDNA | F: GGACTAGTAGGTTCCAATTTTCATTACA  R: CACGGAATGGTTCACGGGTGA |
| myc-GNC Binary | F: GTATGACCGGTATGGAGGAGCAGAAGCTGATC R:CCGGAATTCTCAACCGTGAACCATTCCATAC |
| myc-CGA1 Binary | F: GTATGACCGGGTTCCAATTTTCATTACAC  R: CGTGCCTTAAGTGCCCACTATTCCATAC |
| MYC | F: AATTCGAGGAGCAGAAGCTGATCTCAGAGGAGGACCTGTGAG  R: GATCCTCACAGGTCCTCCTCTGAGATCAGCTTCTGCTCCTCG |
