# Supplementary material for: GNC and CGA1 Modulate Chlorophyll Biosynthesis and Glutamate Synthase (GLU1/Fd-GOGAT) Expression in Arabidopsis
Source: PLoS One. 2011 Nov 10;6(11):e26765. doi: 10.1371/journal.pone.0026765 (PMC3213100; doi:10.1371/journal.pone.0026765)
Supplement: Table S3 — Primers used for semi-quantitative RT- PCR and quantitative Real-time (qRT) PCR analysis of gene expression. (DOC) [file pone.0026765.s004.doc]

| **Table S3.** PCR primers used for semi-quantitative RT- PCR and quantitative Real-time (qRT) PCr analysis of gene expression. | |
| --- | --- |
| Gene Name | Sequence (5´ to 3´) |
| RT-CGA1 | AACCATTCCGTGCGATAGAG  GATACCCATATCTCCCAACCTC |
| RT-GUS | GCGTGACCAAGGAAATCCG  GTTGGGCCATTGAAGTCGG |
| RT-GUS | GCGTGACCAAGGAAATCCG  GTTGGGCCATTGAAGTCGG |
| RT-ARR7 | TGTTCTTGCCGTCGATGATA  CTGCTAGCTTCACCGGTTTC |
| RT- GLU1 | TGGTGGCTGTGTAGTCGTGCT  GCCACAACCTGCTCTTGAATG |
| RT-AHK2 | TGAACCATGTTCATGCCTTG  TTGCCCGTAAGATGTTTTCA |
| RT-AHK3 | ATCAAAGCCTCCCCATTCTT  AACCATTGAGGGCGAGTATG |
| RT-NR2 | CCGACAAGACGGCCAAGTTCGACCT  TCCGTAGAGAAGAACGAAGATCCAAG |
| RT-ACT8 | ATCGTCGTGGTTCTTGTTTCTT  CATCACCAGAGTCCAACACAAT |
| RT-UBQ10 | CTCTCTACCGTGATCAAGATGCA  TGATTGTCTTTCCGGTGAGAGTC |
| qRT-CGA1 | TCCCTCTTCTTTGATGTCACCG  AGTGTGTTGTTTGCTGCTGTCG |
| qRT-GNC | TCCCTCTTCTTTGATGTCACCG  AGTGTGTTGTTTGCTGCTGTCG |
| qRT-NR | ACCGGTTCAATCACTAAACCAT  AATGGAGTGGAACTGGAGTGAT |
| qRT-NiR | ACCGTAGAAAGCATCAGTATG  TCTCTGTAAGCCTCTAGAACA |
| qRT-NRT1 | GCTCTCAAACAAAACAGCCTTT GGCGAATATAGCAATCGTTAGG |
| qRT-NRT2 | TCGTCACTGCCGTTGTATCTAT  ACATGATCAAGCATTGTTGGTT |
| qRT-GLU1 | ATATGCAACGTAGACCGTGCG  ATGAGCCGGATGTTCATACCG |
| qRT-GS1 | CGTCTTCTCTTTCCCTAAACACA  CAGTTCACATCCTTTTGCATCA |
| qRT-GS2 | GCCATCTGTTACAAACACCAAA  TCTGAAGATAGCTTGCGGGTAT |
| qRT-ASN2 | TTCGCGTTTGTCCTTCTTGA  CGAAGCAAACCAGACAGAACC |
| qRT-HEMA1 | GGATCCCACTCGTCTTTGACAT  TGAATGGCCAAGAGCTATTGC |
| qRT-GUN4 | CGGCTTCTCCGGATATCTGAA  GAAACCGCGACCATATTCGAC |
| qRT-PORB | ACCAAATCAAATCCGAACATGG  GGCTCTTTAGCTGTCGGGAAAT |
| qRT-PORC | ACGAGGAAACAGGAGTCACG  GGTCACTCACAACCTGTGCT |
| qRT-ARC3 | CTATGCTGGAAGCTGAACGAGA  ACCGTTGTGCCACATTCCAT |
| qRT-ARC5 | CTGGTGCAGGAGCAATAAGTCC  TGTAGCCCATTCTCTGCAAAGG |
| qRT-ARC6 | GCATTCGCCCATCCAAAAC  CCAGAATGGATGCTAGGACTGC |
| qRT-PDV1 | CTTAACCACGAGCCATCATTACG  CTTAATCACGAGCCATCATTACG |
| qRT-PDV2 | GCATCACGAATACGCAAAAGC GATCTTTCTCCCGGAGAAGGAA |
| qRT-GAPDH | CTTGGAAGGAGCTAGGAATTGACA  ATGTGTTTCCCTGCACCTTCTC |
