# Supplementary material for: GNC and CGA1 Modulate Chlorophyll Biosynthesis and Glutamate Synthase (GLU1/Fd-GOGAT) Expression in Arabidopsis
Source: PLoS One. 2011 Nov 10;6(11):e26765. doi: 10.1371/journal.pone.0026765 (PMC3213100; doi:10.1371/journal.pone.0026765)
Supplement: Table S4 — PCR primers used for ChIP analysis of myc-tagged GNC and CGA1. (DOC) [file pone.0026765.s005.doc]

| **Table S4.** PCR primers used for ChIP analysis of myc-tagged GNC and CGA1. | |
| --- | --- |
| Gene Name | Sequence (5´ to 3´) |

| ChIP-NR-R1 | CGTTGTTCCTATGTTTAACTGAAGG | |
| --- | --- | --- |
| ChIP-NR-F1 | TGTGATCGGTAACTATTAAAACCGTA | |
| ChIP-NR-F2 | CGGCTTTGTGTCACGAATAA | |
| ChIP-NR-R2 | AAAACAAATGTGAATTGTTGGAG | |
| ChIP-NR-F3 | TTTTTGTCAAACAAATGGATGG | |
| ChIP-NR-R3 | AATGCATCGTTGGTGGAGAT | |
| ChIP-NiR-F1 | TCCATTCATAATTGCACACGA | |
| ChIP-NiR-R1 | TGGTTTAGTGCGTGTTTGGA | |
| ChIP-NiR-F2 | TCTCCCTACATGCAAAAATCG | |
| ChIP-NiR-R2 | TCAAATACACTCAGAAGACCAACC | |
| ChIP-NiR-F3 | TGCGGAAACTTGGATGTTAT | |
| ChIP-NiR-R3 | AATAATAAAAGAGATTCGATTTTTGC | |
| ChIP-GLU1- F1 | CCAGCTTGAGCCTCATCAAT | |
| ChIP-GLU1- R1 | AGCACTGGATCTCGTGGTTT | |
| ChIP-GLU1- F2 | GACGGTGAAGAGCAGGAGAG | |
| ChIP-GLU1- R2 | CATTGGGGGTTTAGGGTTTT | |
| ChIP-GLU1- F3 | CTGAAGAAGCGCCATGAAAT | |
| ChIP-GLU1- R3 | CCTACTGTTGGGCTTTGGAA | |
| ChIP-HEMA1-R1 | | AGAATTTTGAGAATACCAACAATATTTAA |
| ChIP-HEMA1-F1 | | TCTGATGAGGAAATAAATTGGAAA |
| ChIP-HEMA1-R2 | | CGTCTTAGGTACATAATCTTTTGCTTA |
| ChIP-HEMA1-F2 | | TCATGAAGCAACCGATGGA |
| ChIP-HEMA1-R3 | | CAATTTTTTTTTTTGTTAAAGCAGA |
| ChIP-HEMA1-F3 | | TTGTTGGTATTCTCAAAATTCTTCTTA |
| ChIP-GUN4-R1 | | AAACTTACAGAGCAGTGATCTCCTCA |
| ChIP-GUN4-F1 | | ACGAGGAGGTAGCGGAAGAAG |
| ChIP-GUN4-R2 | | GCGCCATCAGAGTTCTATGTTG |
| ChIP-GUN4-F2 | | AACTCATTGGCGTATAAATAGGCC |
| ChIP-GUN4-R3 | | TTATAACAGTGTTTTGACCGATATTGATT |
| ChIP-GUN4-F3 | | TCTCTCGTTATCGTTATTGTTTGAAAG |
| ChIP-PORB-R1 | | ATGTTGTTTTGTTGGCGAAA |
| ChIP-PORB-F1 | | CTGAGAGACAATGGCGTTGA |
| ChIP-PORB-F2 | | TCAACGCCATTGTCTCTCAG |
| ChIP-PORB-R2 | | TTGTACATAGAAAACCCACCTCAA |
| ChIP-PORB-F3 | | ACACCAATCAGCAATCACCA |
| ChIP-PORB-R3 | | TTCGGACAAAGGAAGATTGG |
| ChIP-PORC-F1 | | GGTTGCGTACAGGTCAGAGC |
| ChIP-PORC-R1 | | GGAAATGATGATGGAACTTTTGA |
| ChIP-PORC-F2 | | TTTCTCCCACCTCTCCATTG |
| ChIP-PORC-R2 | | CACGTGTCTTCCAGGCTACA |
| ChIP-ARC3-R1 | | AAGGATACGTTTCGGGTCGG |
| ChIP-ARC3-F1 | | CCTTTTCGCACTCAGCGAAT |
| ChIP-ARC3-R2 | | CAGATGTTTTTATTAATTATTTCGATGTCA |
| ChIP-ARC3-F2 | | TTCTTTCTTAACAACAACTAGAGAAAACAATTT |
| ChIP-ARC3-R3 | | TATATCATTGACCAAGAAAGTAAACTATTAAAAGT |
| ChIP-ARC3-F3 | | GACGAATCGGTGGCGG |
| ChIP-ARC5-R1 | | TCAAGCGCCACAAACCG |
| ChIP-ARC5-F1 | | CGAGGGCTTGTTCTGGCTC |
| ChIP-ARC5-R2 | | TCGATGAAAGTCTGTGCTATTCAAC |
| ChIP-ARC5-F2 | | AAACCTAAAGACAATTACAAAATGTGTATACA |
| ChIP-ARC5-R3 | | AATATCTACTGACAGAAAATCTAATTCTCGAA |
| ChIP-ARC5-F3 | | CCTCCCATATGTGTTTCCTAGGA |
| ChIP-ARC6-R1 | | TATTGGGTTTTTGGCTTTGC |
| ChIP-ARC6-F1 | | TGGCACACTCGATTGGTTTA |
| ChIP-ARC6-R2 | | TCGAGTGTGCCAGTGAAATC |
| ChIP-ARC6-F2 | | ATGTTGGAGCAGAGCTTCGT |
| ChIP-ARC6-R3 | | GAGGGAATTGATGGGGTTTT |
| ChIP-ARC6-F3 | | GTCGCCACGGACTTTATGAT |
| ChIP-PDV1-F1 | | TGGTTTCTACCGGTTTACGG |
| ChIP-PDV1-R1 | | TTCTACCGCGGTGATAAAATG |
| ChIP-PDV1-F2 | | TGGGAGAGGCTAAAGTGCTC |
| ChIP-PDV1-R2 | | GCCGACGTGTCAAATGTAAA |
| ChIP-PDV1-F3 | | TGGGAGAGGCTAAAGTGCTC |
| ChIP-PDV1-R3 | | GCCGACGTGTCAAATGTAAA |
| ChIP-PDV2-R1 | | AATCTGATTCCCAACAATTGGTG |
| ChIP-PDV2-F1 | | TCTGCCACTCCATTGCCTC |
| ChIP-PDV2-R2 | | TGTCAGATTTGTCATCTTTGTTAGAGTC |
| ChIP-PDV2-F2 | | TTTCTGCTGCTTTCCCGGT |
| ChIP-PDV2-R3 | | AGAGAATTACGCTGTCATATATACCAAGTATAA |
| ChIP-PDV2-F3 | | CGAAGTCAACTGATCGGTTTACC |
